# Synthesis and late stage modifications of Cyl derivatives

**DOI:** 10.3762/bjoc.18.19

**Published:** 2022-02-04

**Authors:** Phil Servatius, Uli Kazmaier

**Affiliations:** 1 Organic Chemistry, Saarland University, Campus C4.2, 66123 Saarbrücken, Germany

**Keywords:** chelated enolate, Claisen rearrangement, HDAC inhibitor, peptide, late stage modification

## Abstract

A peptide Claisen rearrangement is used as key step to generate a tetrapeptide with a C-terminal double unsaturated side chain. Activation and cyclization give direct access to cyclopeptides related to naturally occurring histone deacetylase (HDAC) inhibitors Cyl-1 and Cyl-2. Late stage modifications on the unsaturated amino acid side chain allow the introduction of functionalities which might coordinate to metal ions in the active center of metalloproteins, such as histone deacetylases.

## Introduction

**Figure 1:**
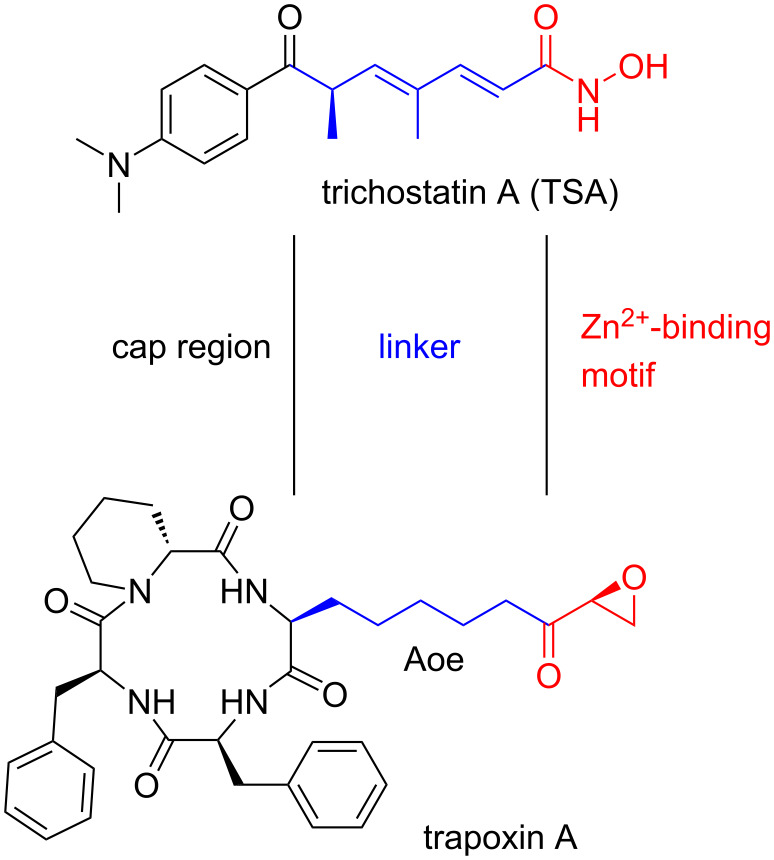
Naturally occurring HDAC inhibitors.

Among natural products, peptidic structures have entered the limelight due to their extraordinary biological activities [1]. Often found as secondary metabolites for self-defense in different microorganisms, peptidic natural products are assembled either by ribosomal synthesis or by non-ribosomal peptide synthetases (NRPS) [2]. Macrocyclic peptides are pervasive throughout this class of natural products and often show improved stability against proteolytic digest and metabolic processes [3]. Furthermore, cyclization generally helps to fix the active conformation of a peptide needed to interact with the respective cellular target. Incorporation of non-proteinogenic and unusual amino acids often is related to their biological function. For example, trapoxin B (Figure 1) is a cyclic tetrapeptide with a rather unusual epoxyketone side chain and was found to be a strong inhibitor of histone deacetylases (HDACs) [4–5].

HDACs are nuclear isozymes that regulate gene transcription via a dynamic process of acetylation and deacetylation of lysine residues of histones [6–10]. Blockade of the deacylating process causes hyperacetylation of histones and unregulated gene activity, that results in untimely cell death. Eighteen different HDAC enzymes are known so far and they are divided into four classes based on structural homology with yeast proteins [11]. Three of these enzyme classes (I, II, and IV) contain Zn^2+^ within the active site, and therefore these enzymes can be affected by typical Zn^2+^-binding HDAC inhibitors. In cellular systems, an acetylated lysine of a histone is entering the cavity of the active site and gets coordinated to Zn^2+^. Subsequent attack of water forms a tetrahedral intermediate which results in a cleavage of the acetylated lysine. Most HDAC inhibitors act as substrate mimics and contain a zinc-binding motif. They competitively interact with the HDACs to form stable intermediates and therewith block the active site.

Many naturally occurring HDAC inhibitors are known to date [12]. Acyclic molecules like, e.g., trichostatin A (TSA, Figure 1) were among the first isolated HDAC inhibitors. Isolated in 1976 from *Streptomyces hygroscopicus* by Tsuji et al. [13], TSA played an important role in rationalizing the mode of action of HDACs [14]. Trichostatin contains a hydroxamic acid as zinc-binding motif, inspiring the design of a wide range of synthetic HDAC inhibitors. The essential Zn^2+^-binding group is attached to a non-polar linker, delivering it inside the cavity through a narrow channel. The cap region is responsible for interactions with residues on the rim of the active site [15]. The cap region of acyclic HDAC inhibitors is generally small, resulting in non-specific interactions with the different HDAC isoforms. More diverse cap regions are found in macrocyclic HDAC inhibitors such as trapoxin which contains the unusual, non-proteinogenic amino acid (2*S*,9*S*)-2-amino-9,10-epoxy-8-oxodecanoic acid (Aoe) as a zinc-binding group.

**Figure 2:**
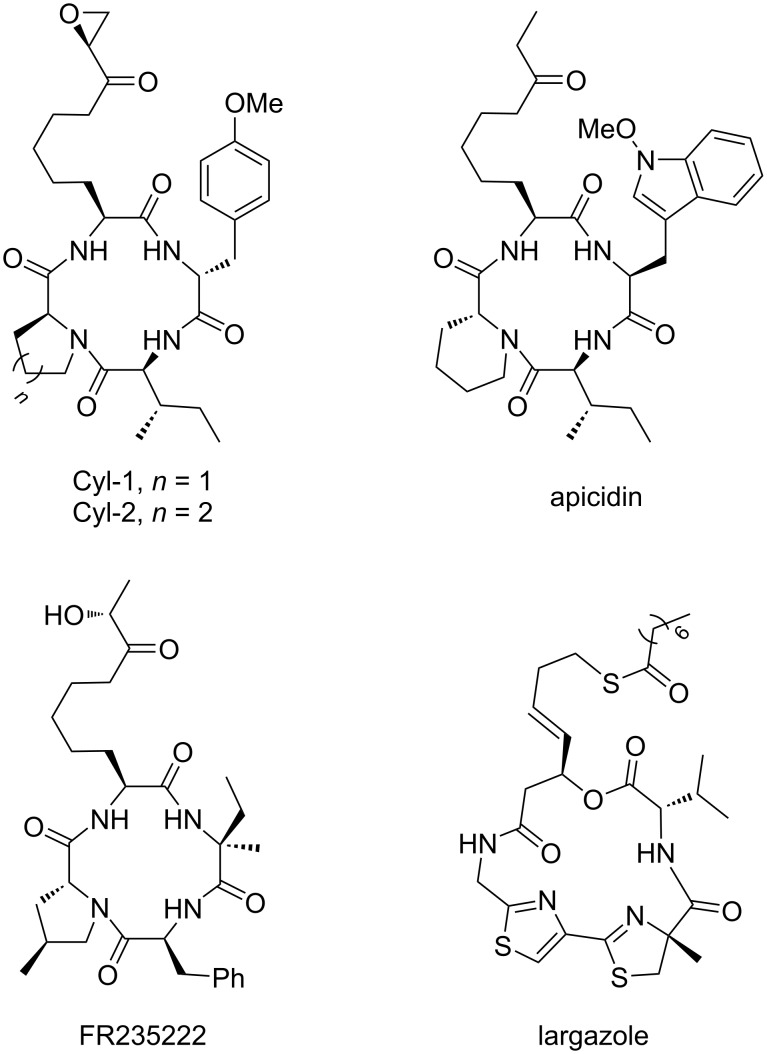
Naturally occurring HDAC inhibitors with different zinc-binding motifs.

Interestingly, Aoe with its α-epoxyketone motif is wide-spread among this compound class as it is present in other natural products such as Cyl-1 and Cyl-2 [16–17], chlamydocin [18], and many others [12]. The α-epoxyketone is isosteric to an acetylated lysine residue, which makes it a mimic of HDAC’s natural substrate [10]. Although α-epoxyketones and hydroxamic acids show high affinities towards Zn^2+^, other chelating groups are also found in natural products (Figure 2) including ketones (apicidin [19], microsporin A [20]), carboxylic acids (azumamides [21]), α-hydroxy ketones (FR235222 [22]) or thioesters (largazole [23]). These cyclopeptides mainly differ in the amino acid sequence of the peptide backbone, which causes selectivity towards the different HDAC isoforms. In fact, many naturally occurring HDAC inhibitors contain sulfur moieties like, e.g., disulfides or thioesters. They seem to lack a zinc-chelating group at first sight, but the disulfide or thioester acts as a prodrug and are reduced/cleaved in vivo to liberate the free thiol, a strong Zn-binding group [24–25].

## Results and Discussion

Since a couple of years the focus of our research group is on the synthesis of unnatural amino acids and their incorporation into complex natural products. Many of these show interesting anticancer activities [26–28]. Some linear peptides, such as pretubulysin bind to tubulin [29–31], while others cyclodepsipeptides are strong actin binders [32–33]. Recently, we also became interested in the synthesis of the cyclic HDAC inhibitors. We developed syntheses for chlamydocin [34], Cyl-1 [35], and trapoxin [36] using either Claisen rearrangements [37–38] or Pd-catalyzed allylic alkylations [39–40] as key steps for the synthesis of the unusual Aoe, which was then incorporated into the different tetrapeptides.

Our aim now was to develop a rather flexible protocol that allows us to introduce several types of functionalities onto a given peptide to create libraries of structurally related compounds for SAR studies. Of course, this approach is not limited to the development of HDAC inhibitors, but should be suitable for all kinds of natural product modifications. However, the structural motif of the natural products shown in Figure 2 is suitable to illustrate the concept.

**Scheme 1:**
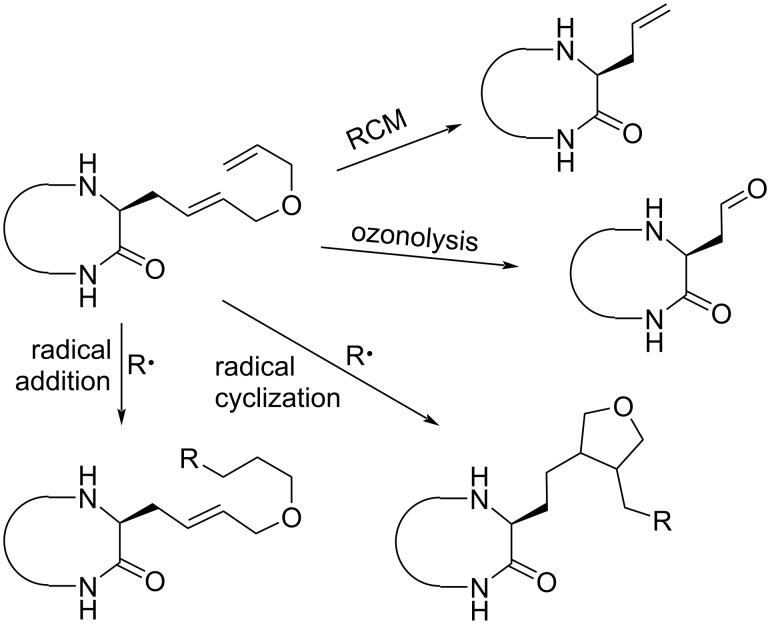
Planned syntheses of Cyl-1 derivatives.

In general, typical syntheses of such natural products start with the synthesis of the unusual building blocks, their incorporation into a linear peptide, which is finally subjected to cyclization at a suitable position. No question, this protocol is well suited to get access to a certain compound, also in large scale, but it is not applicable for the generation of small libraries of related compounds for SAR studies. Therefore, it is more convenient to undertake modifications at a late stage of the synthesis using a suitably modified precursor allowing variations in a straightforward manner. As a model compound, we decided to use the Cyl-1 amino acid backbone and introduce a double unsaturated side chain (Scheme 1). In principle, selective modifications at the two different double bonds (internal and terminal) should be possible. Ring-closing metathesis (RCM) should generate an allylglycine unit, which should undergo a wide range of addition reactions. Ozonolysis, on the other hand, should generate a carbonyl functionality. Radical additions towards the double unsaturated side chain of the Cyl-1 derivative might also allow cyclizations.

**Scheme 2:**
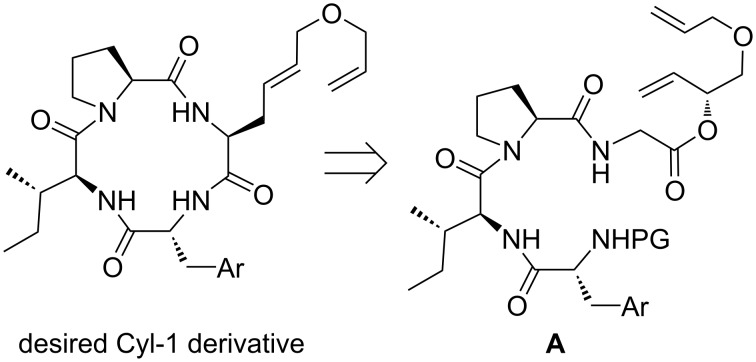
Cyl-1 derivatives via peptide Claisen rearrangement.

To get access to the desired double unsaturated cyclopeptide, we decided to take advantage of an asymmetric chelate enolate Claisen rearrangement, which should allow the stereoselective generation of the unusual amino acid, depending on the configuration of the chiral allylic alcohol used [41–42]. If a peptide Claisen rearrangement [43–45] is carried out with a suitable protected linear precursor **A** (Scheme 2, PG: protecting group), the resulting carboxylic acid obtained can directly be activated and subjected to cyclization. If the glycine allyl ester is incorporated as the last building block into the C-terminus of the peptide, this concept should provide a high degree of variability for the generation of small libraries, in our case of Cyl-1 derivatives.

**Scheme 3:**
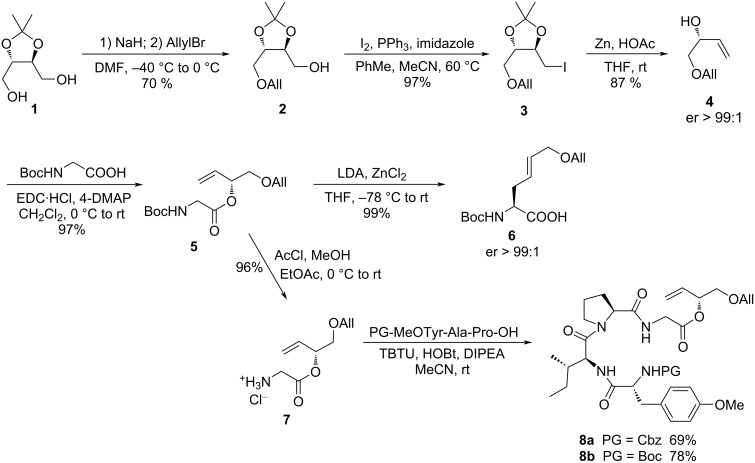
Synthesis of tetrapeptide allyl esters **8**.

Chiral allylic alcohols are easily accessible, either via kinetic resolution of racemic alcohols [46–47], asymmetric catalysis [48], or from chiral pool materials, such as threitol **1** [49]. Using the last approach, **1** was mono-*O*-allylated to **2** under similar conditions reported previously for monobenzylation (Scheme 3) [50]. Iodination (**3**) and subsequent elimination of the iodide with zinc dust gave allylic alcohol **4** as a single enantiomer, which was esterified with Boc-protected glycine to allyl ester **5**. Before we incorporated this allylic ester into the desired tetrapeptide, we wanted to make sure that the chelate Claisen rearrangement does not cause any problems. And indeed, Claisen rearrangement of **5** proceeded cleanly, providing the protected amino acid **6** in almost quantitative yield and with perfect chirality transfer.

With this positive results in hand, we incorporated **5** into the desired tetrapeptide **8**. So far, we carried out peptide Claisen rearrangements only with small dipeptides, but never used longer peptide chains, such as tetrapeptides. We knew from previous work that the protecting groups on the peptide can have a significant effect on the Claisen rearrangement and therefore we synthesized the Cbz- as well as the Boc-protected peptides **8a** and **8b**. The tripeptide building blocks were previously also used in the Cyl-1 synthesis. Glycine allyl ester **5** was Boc-deprotected to give amine **7** as hydrochloride salt, using a protocol developed by Nudelman et al. [51]. Coupling with the protected tripeptides using TBTU occurred without epimerization [52].

The two tetrapeptide allyl esters were subjected to the peptide Claisen rearrangement, the key step of the synthesis. Subjecting allyl ester **8a** to the usual conditions of an ester enolate Claisen rearrangement with zinc chloride as chelating metal gave the rearranged product in only 33% yield and a diastereomeric ratio of 93:7 (Table 1, entry 1). The reaction was kept at −45 °C overnight to suppress potential epimerization of the peptide. Generally, epimerization is prevented through deprotonation of amide NH bonds, as argued by Seebach for Li enolates [53–54]. Nevertheless, isoleucine was prone to epimerize under the reaction conditions due to its vicinity to proline and therewith lack of the “protecting” NH group. Since no full conversion was observed in this first attempt, LDA was replaced with LHMDS and the reaction was allowed to warm to room temperature overnight (Table 1, entry 2). LHMDS is a weaker base than LDA and should not deprotonate α-substituted amino acid amides [53–54]. No full conversion was observed either and both yield and diastereomeric ratio were similar to the reaction with LDA. If the Boc-protected ester **8b** was then treated with LHMDS under the same reaction conditions (Table 1, entry 3) surprisingly no conversion was observed at all. Switching the base back to LDA (Table 1, entry 4) gave similar results than before (Table 1, entry 1). Since the reaction seemed to stop after 30–40% conversion, it was speculated that the ester enolate chelate complex formation was incomplete due to consumption of the base. For instance, deprotonation of tyrosine residues in benzyl position has been observed previously in the derivatization of miuraenamides and would call for an additional equivalent of base. Therefore, the reaction was repeated with 5.5 equiv LDA (Table 1, entry 5) and indeed, the desired tetrapeptide acid was obtained in quantitative yield as crude product, without obvious formation of byproducts. Strikingly, the diastereomeric ratio was also very high. Only prolonged reaction times (>18 h) led to epimerization of the rearranged peptide.

**Table 1:** Ester enolate Claisen rearrangements of tetrapeptide allyl esters **8**.

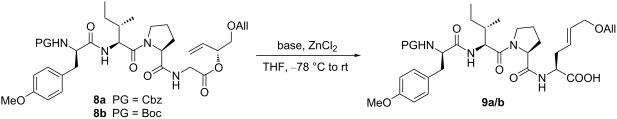						

entry	**8**	base (equiv)	**9**	yield [%]	dr	comment

1	**8a**	LDA (4.8)	**9a**	33	93:7	no full conversion
2	**8a**	LHMDS (4.8)	**9a**	37	≈95:5	no full conversion
3	**8b**	LHMDS (4.5)	**9b**	–	–	no conversion
4	**8b**	LDA (4.5)	**9b**	34	95:5	no full conversion
5	**8b**	LDA (5.5)	**9b**	quant.	>99:1	full conversion

**Scheme 4:**
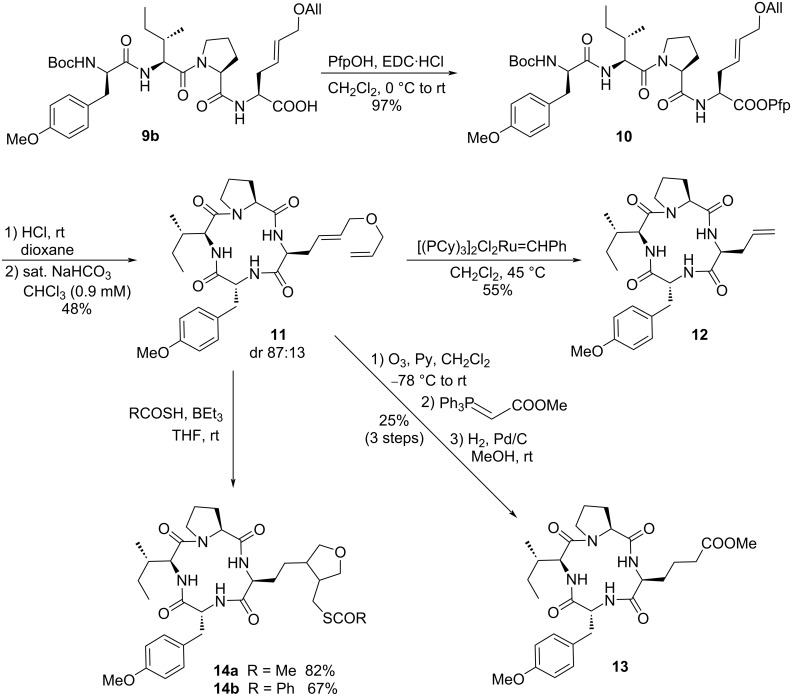
Synthesis and late stage modifications of Cyl derivatives.

With rearranged tetrapeptide **9b** in hand, esterification with pentafluorophenol (Pfp) gave Pfp ester **10**, which should readily cyclize after Boc deprotection (Scheme 4). Treatment with HCl in dioxane gave the crude ammonium salt, which was subjected to biphasic ring closure; the hydrochloride salt was added dropwise to a stirred emulsion of saturated NaHCO_3_ solution in chloroform [55]. Macrocycle **11** was obtained in acceptable yield as a diastereomeric mixture (dr 87:13), but the diastereomers of **11** could be separated by reversed-phase flash chromatography. It was obviously the C-terminal unusual amino acid which underwent epimerization under the reaction conditions, since already the Pfp ester **10** was partially epimerized, as determined later on. To access the allylglycine motif for further derivatization, pure cycle **11** was subjected to Grubbs I catalyst in dichloromethane at 45 °C to get the desired product **12**. While the reaction proceeded well even with the cyclic tetrapeptide, compound **12** proved to be highly insoluble, which complicated its purification.

An acknowledged method for the removal of metathesis catalysts is the formation of Ru-DMSO complexes, which do not eluate from a silica column [56]. This allowed us to remove at least the Ru contamination, but we were unable to subject **12** to further modifications such as cross metathesis or thiol-ene click reactions due to poor solubility. Additionally, providing sophisticated NMR spectra of **12** turned out to be a non-trivial issue. All commercially available deuterated solvents were tested as solvents and finally, recording the spectra in tetrachloroethane-*d*_2_ at elevated temperatures (100 °C) led to a clear solution and hence clean NMR spectra.

The solubility issues forced us to investigate also other modification protocols. Thus, macrocycle **11** was subjected to an ozonolysis with subsequent Wittig reaction in a one-pot manner (Scheme 4). Performing the ozonolysis in presence of pyridine led to immediate reduction of the primary ozonide formed during the reaction [57]. Consequently, no PPh_3_ or Me_2_S was required to obtain the crude aldehyde. Subsequent addition of a Wittig ylide gave access to a cyclopeptide with an α,β-unsaturated ester side chain as a (*E*/*Z*) mixture. Unfortunately, this compound contained triphenylphosphine oxide as impurity, which could not be separated from the product. Subsequent hydrogenation proceeded readily and afforded the saturated cyclopeptide **13**. However, the impurity could also not be removed on this stage. Apparently, the Cyl derivatives with a short side chain are not good candidates for further modifications, mainly for solubility reasons.

Therefore, we decided to have a closer look into modifications of the longer side chain present in **11** and subjected it to thiol-ene click reactions. Since masked thiols are often found as zinc-coordinating functionalities in HDAC inhibitors, e.g., in the largazoles, we treated **11** with thioacetic acid and BEt_3_/air in THF to give 82% of the thiol-ene click product (Scheme 4). Careful analysis of the NMR spectra revealed that the intermediately formed radical cyclized in an intramolecular 5-*exo*-*trig* fashion with the internal double bond to form a tetrahydrofuran cycle **14a**. The formation of the 5-membered ring system seemed to be the driving force of this reaction [58]. Comparable yields were obtained with other thiocarboxylic acids, such as thiobenzoic acid, which gave rise to the benzoylated cyclopeptide **14b**. In these cases, the obtained products were nicely soluble and could easily be purified.

## Conclusion

In conclusion, we could show that chelate Claisen rearrangements can be carried out in longer peptides, such as tetrapeptides, as long as acidic positions can be identified, and the amount of base can be adjusted accordingly. The formation of thiol-ene click products **14** substantiated the hypothesis that the insolubility of Cyl derivatives with short side chains limit their synthetic applicability. The fact that **11** underwent rapid intramolecular cyclization after thiol addition renders further investigations into thiol-ene click-initiated cyclization reactions. The chain length in **14** should generally be suitable for effective HDAC inhibition and the thioester moiety might act as a prodrug as described for the natural HDAC inhibitor largazole. Further investigations are currently in progress.

## Supporting Information

File 1 Detailed synthetic procedures, characterization of all molecules and copies of NMR spectra.
