# Supplementary material for: Synthesis and late stage modifications of Cyl derivatives
Source: Beilstein J Org Chem. 2022 Feb 4;18:174–81. doi: 10.3762/bjoc.18.19 (PMC8822456; doi:10.3762/bjoc.18.19)

BEILSTEIN JOURNAL OF ORGANIC CHEMISTRY

Supporting Information

for

Synthesis and late stage modifications of Cyl derivatives

Phil Servatius and Uli Kazmaier

Beilstein J. Org. Chem. **2022,** *18,* 174–181. doi:10.3762/bjoc.18.19

Detailed synthetic procedures, characterization of all molecules and copies of NMR spectra

License and Terms: This is a supporting information file under the terms of the Creative Commons Attribution License (https://creativecommons.org/licenses/by/4.0). Please note that the reuse, redistribution and reproduction in particular requires that the author(s) and source are credited and that individual graphics may be subject to special legal provisions.
The license is subject to the *Beilstein Journal of Organic Chemistry* terms and conditions: (https://www.beilstein-journals.org/bjoc/terms)

Table of contents

Experimental S2

References S14

Copies of NMR spectra S15

Experimental

General remarks. All air- or moisture-sensitive reactions were carried out in dried glassware (> 100 °C) under an atmosphere of nitrogen or argon. THF was distilled over Na/benzophenone prior to use. Ethyl acetate (EtOAc) and petroleum ether (petroleum ether) were distilled prior to use. Reactions were monitored by analytical TLC, which was performed on precoated silica gel on TLC PET-foils. Visualization was accomplished with UV-light (254 nm), $KMnO_4$ solution or Ce(IV) solution. The products were purified by flash chromatography on silica gel columns (0.063–0.2 mm) or by automated flash chromatography. Mixtures of EtOAc and petroleum ether were generally used as eluents. Melting points were determined with a melting point apparatus and are uncorrected. ^{1}H and ^{13}C NMR spectra were recorded with a 400 MHz (100 MHz) or 500 MHz (125 MHz) spectrometer in $CDCl_3$ unless otherwise specified. Chemical shifts are reported in ppm relative to $Si(CH_3)_4$ and $CHCl_3$ was used as the internal standard. Multiplicities are reported as br (broad signal), s (singlet), d (doublet), t (triplet), q (quartet) and m (multiplet). Mass spectra were recorded with a high resolution quadrupole spectrometer (CI) or a UHR-TOF-spectrometer using the ESI technique. Regio- and stereoisomeric ratios were determined by GC equipped with a CP-Chirasil-Dex CB capillary column (25 m × 0.25 mm) using nitrogen as carrier gas. HPLC analyses were performed using a Diode Array detector. Optical rotations were measured in a thermostated (20 °C ± 1 °C) cuvette. The radiation source used was a sodium vapor lamp (λ = 589 nm). The concentrations are given in g/100 mL.

((4*S*,5*S*)-5-((Allyloxy)methyl)-2,2-dimethyl-1,3-dioxolan-4-yl)methanol (2)

Sodium hydride (2.20 g, 55.0 mmol, 60 wt % in paraffin) was placed in a three-necked flask and 10 mL *n*-pentane were added. After stirring for a few minutes, the sodium hydride was allowed to segregate and the solvent was removed *via* syringe. The procedure was repeated two times and the oil-free NaH was dried *in high-vacuo*.

The free sodium hydride was suspended in 35 mL DMF abs. and cooled to −40 °C. A solution of 8.15 g (50.3 mmol) (+)-2,3-*O*-isopropylidene-L-threitol (**1**) in 45 mL DMF abs. was added carefully within 50 min. Afterwards, a solution of 4.46 mL (6.24 g, 51.6 mmol) allyl bromide in 45 mL DMF abs. was added dropwise within 20 min at −40 °C and the reaction was allowed to warm to 0 °C slowly. The solvent was then distilled off *in vacuo* (0.43 mbar, 30–50 °C oil bath). The residue was dissolved in dichloromethane and water was added. The layers were separated and the aqueous phase was extracted three times with dichloromethane. The combined organic layers were washed with brine and dried over Na_2SO_4. The crude product was purified by column chromatography (silica gel, petroleum ether/ethyl acetate 80:20, 70:30). 7.12 g (35.2 mmol, 70%) of compound **2** were obtained as a colorless oil. R_f (**2**) = 0.32 (silica gel, petroleum ether/ethyl acetate 50:50). $[\alpha]_D^{20}$ = +3.5 (*c* = 1.0, $CHCl_3$).

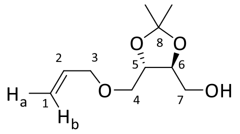

^{1}H NMR (400 MHz, $CDCl_3$): δ = 1.41 (s, 6 H, 9-H, 9'-H), 2.28 (dd, $^{3}J_{OH,7a}$ = 8.2 Hz, $^{3}J_{OH,7b}$ = 4.6 Hz, 1 H, OH), 3.52 (dd, $^{2}J_{4a,4b}$ = 9.9 Hz, $^{3}J_{4a,5}$ = 5.6 Hz, 1 H, 4-H_a), 3.65 (dd, $^{2}J_{4b,4a}$ = 9.9 Hz, $^{3}J_{4b,5}$ = 5.1 Hz, 1 H, 4-H_b), 3.71 (ddd, $^{2}J_{7a,7b}$ = 11.9 Hz, $^{3}J_{7a,OH}$ = 8.2 Hz, $^{3}J_{7a,6}$ = 4.2 Hz, 1 H, 7-H_a), 3.78 (ddd, $^{2}J_{7b,7a}$ = 11.6 Hz, $^{3}J_{7b,OH}$ ≈ $^{3}J_{7b,6}$ = 4.5 Hz, 1 H, 7-H_b), 3.93 (dt, $^{3}J_{6,5}$ = 8.4 Hz, $^{3}J_{6,7}$ = 4.2 Hz, 1 H, 6-H), 4.02 (m, 1 H, 5-H), 4.04 (d, $^{3}J_{3,2}$ = 5.5 Hz, 2 H, 3-H), 5.20 (ddt, $^{3}J_{1a,2}$ =

10.4 Hz, $^2J_{1a,1b}$ ≈ $^4J_{1a,3}$ = 1.3 Hz, 1 H, 1-H$_a$), 5.27 (ddt, $^3J_{1b,2}$ = 17.2 Hz, $^2J_{1b,1a}$ ≈ $^4J_{1b,3}$ = 1.6 Hz, 1 H, 1-H$_b$), 5.89 (dddd, $^3J_{2,1b}$ = 17.1 Hz, $^3J_{2,1a}$ = 10.4 Hz, $^3J_{2,3a}$ = 5.8 Hz, $^3J_{2,3b}$ = 5.5 Hz, 1 H, 2-H) ppm. ^{13}C NMR (100 MHz, $CDCl_3$): δ = 26.9 (q, C-9), 26.9 (q, C-9'), 62.4 (t, C-7), 70.4 (t, C-4), 72.6 (t, C-3), 76.6 (d, C-5), 79.6 (d, C-6), 109.3 (s, C-8), 117.6 (t, C-1), 134.1 (d, C-2) ppm. HRMS (CI) calculated for $C_{10}H_{19}O_4$ [M + H]$^+$: 203.1278, found: 203.1283.

(4*S*,5*R*)-4-((Allyloxy)methyl)-5-(iodomethyl)-2,2-dimethyl-1,3-dioxolane (3)

To a solution of 7.01 g (34.7 mmol) **2** in 100 mL toluene abs. and 20 mL MeCN abs. was added 2.95 g (43.3 mmol) imidazole, 9.55 g (36.4 mmol) PPh_3 and 9.23 g (36.4 mmol) iodine carefully at room temperature. The reaction mixture was heated to 60 °C for 2 h. For workup, the reaction mixture was diluted with ether and washed with sat. $Na_2S_2O_3$ sol., water, and brine. The organic phase was dried over Na_2SO_4 and concentrated *in vacuo*. Purification by column chromatography (silica gel, *n*-pentane/ether 2:1) afforded 10.5 g (33.6 mmol, 97%) of iodide **3** as a colorless liquid. R$_f$ (**3**) = 0.25 (silica gel, petroleum ether/ethyl acetate 90:10). $[\alpha]_D^{20}$ = −11.6 (*c* = 1.0, $CHCl_3$).

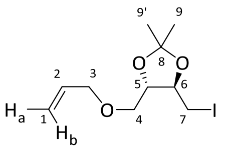

^{1}H NMR (400 MHz, $CDCl_3$): δ = 1.42 (s, 3 H, 9-H), 1.47 (s, 3 H, 9'-H), 3.29 (dd, $^2J_{7a,7b}$ = 10.5 Hz, $^3J_{7a,6}$ = 5.3 Hz, 1 H, 7-H$_a$), 3.36 (dd, $^2J_{7b,7a}$ = 10.5 Hz, $^3J_{7b,6}$ = 5.3 Hz, 1 H, 7-H$_b$), 3.60 (dd, $^2J_{4a,4b}$ = 10.3 Hz, $^3J_{4a,5}$ = 4.9 Hz, 1 H, 4-H$_a$), 3.64 (dd, $^2J_{4b,4a}$ = 10.3 Hz, $^3J_{4b,5}$ = 5.3 Hz, 1 H, 4-H$_b$), 3.85 (dt, $^3J_{6,5}$ = 7.5 Hz, $^3J_{6,7}$ = 5.3 Hz, 1 H, 6-H), 3.95 (dt, $^3J_{5,6}$ = 7.5 Hz, $^3J_{5,4}$ = 5.3 Hz, 1 H, 5-H), 4.05 (d, $^3J_{3,2}$ = 5.6 Hz, 2 H, 3-H), 5.20 (ddt, $^3J_{1a,2}$ = 10.4 Hz, $^2J_{1a,1b}$ = 2.9 Hz, $^4J_{1a,3}$ = 1.3 Hz, 1 H, 1-H$_a$), 5.28 (ddt, $^3J_{1b,2}$ = 17.2 Hz, $^2J_{1b,1a}$ = 3.3 Hz, $^4J_{1b,3}$ = 1.6 Hz, 1 H, 1-H$_b$), 5.90 (dddd, $^3J_{2,1b}$ = 17.3 Hz, $^3J_{2,1a}$ = 10.5 Hz, $^3J_{2,3a}$ = 5.8 Hz, $^3J_{2,3b}$ = 5.5 Hz, 1 H, 2-H) ppm. ^{13}C NMR (100 MHz, $CDCl_3$): δ = 6.3 (t, C-7), 27.3 (q, C-9), 27.3 (q, C-9'), 70.6 (t, C-4), 72.6 (t, C-3), 77.6 (d, C-6), 80.1 (d, C-5), 109.8 (s, C-8), 117.4 (t, C-1), 134.3 (d, C-2) ppm. HRMS (CI) calculated for $C_{10}H_{18}IO_3$ [M+H]$^+$: 313.0295, found: 313.0312.

(*R*)-1-(Allyloxy)but-3-en-2-ol (4)

A solution of 10.5 g (33.6 mmol) **3** in 135 mL THF abs. was treated with 8.78 g (134 mmol) zinc dust and 9.60 mL (10.1 g, 168 mmol) acetic acid at room temperature and the reaction mixture was stirred for 3 h. The reaction mixture was filtrated through a pad of Celite® and washed with ether. The filtrate was concentrated *in vacuo* and the residue was dissolved in ether, sat. $NaHCO_3$ sol. was added and the layers were separated. The aqueous phase was extracted three times with diethyl ether and the combined organic layers were dried over Na_2SO_4. Purification by column chromatography (silica gel, petroleum ether/ethyl acetate 80:20, 70:30) gave 3.76 g (29.3 mmol, er > 99:1, 87%) allylic alcohol **4** as a slightly yellow liquid. R$_f$ (**4**) = 0.21 (silica gel, petroleum ether/ethyl acetate 70:30). $[\alpha]_D^{20}$ = −1.6 (*c* = 1.0, $CHCl_3$). GC: CP-Chirasil-Dex CB, column flow 1.25 mL/min, injector 250 °C; 60 °C (5 min), 80 °C (1 °C/min), 5 min, 170 °C (6 °C/min), 10 min: (*R*)-**4**: t$_R$ = 31.51 min (> 99%).

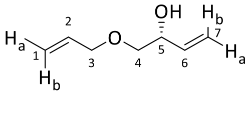

^{1}H NMR (400 MHz, $CDCl_3$): δ = 2.65 (br s, 1 H, OH), 3.34 (dd, $^2J_{4a,4b}$ = 9.7 Hz, $^3J_{4a,5}$ = 7.9 Hz, 1 H, 4-H_a), 3.51 (dd, $^2J_{4b,4a}$ = 9.7 Hz, $^3J_{4b,5}$ = 3.4 Hz, 1 H, 4-H_b), 4.04 (ddd, $^3J_{3,2}$ = 5.8 Hz, $^4J_{3,1a}$ ≈ $^4J_{3,1b}$ = 1.3 Hz, 2 H, 3-H), 4.33 (m, 1 H, 5-H), 5.20 (m, 2 H, 1-H_a, 7-H_a), 5.28 (ddt, $^3J_{1b,2}$ = 17.3 Hz, $^2J_{1b,1a}$ = 1.8 Hz, $^4J_{1b,3}$ = 1.5 Hz, 1 H, 1-H_b), 5.36 (ddd, $^3J_{7b,6}$ = 17.3 Hz, $^2J_{7b,7a}$ ≈ $^4J_{7b,5}$ = 1.5 Hz, 1 H, 7-H_b), 5.84 (ddd, $^3J_{6,7b}$ = 17.2 Hz, $^3J_{6,7a}$ = 10.5 Hz, $^3J_{6,5}$ = 5.5 Hz, 1 H, 6-H), 5.91 (dddd, $^3J_{2,1b}$ = 17.3 Hz, $^3J_{2,1a}$ = 10.5 Hz, $^3J_{2,3a}$ = 5.8 Hz, $^3J_{2,3b}$ = 5.5 Hz, 1 H, 2-H) ppm. ^{13}C NMR (100 MHz, $CDCl_3$): δ = 71.5 (d, C-5), 72.2 (t, C-3), 73.9 (t, C-4), 116.4 (t, C-7), 117.4 (t, C-1), 134.3 (d, C-2), 136.5 (d, C-6) ppm. HRMS (CI) calculated for $C_7H_{13}O_2$ [M + H]$^+$: 129.0910, found: 129.0908.

(*R*)-1-(Allyloxy)but-3-en-2-yl (*tert*-butoxycarbonyl)glycinate (5)

Boc-Gly-OH (990 mg, 5.65 mmol) and 658 mg (5.13 mmol) **4** were dissolved in 20.0 mL dichloromethane abs. before 62.4 mg (0.511 mmol) 4-DMAP and 1.18 g (6.16 mmol) EDC·HCl were added at 0 °C. The reaction mixture was stirred at this temperature for 1 h and afterwards at room temperature for 30 min. For workup, the solution was diluted with dichloromethane and 1 M HCl sol. was added. The layers were separated and the aqueous phase was extracted three times with dichloromethane. The combined organic phases were washed with sat. $NaHCO_3$, dried over Na_2SO_4 and concentrated *in vacuo*. 1.42 g (4.98 mmol, 97%) of ester **5** were obtained as a colorless oil. R_f (**5**) = 0.43 (silica gel, petroleum ether/ethyl acetate 60:40). $[\alpha]_D^{20}$ = −3.8 (*c* = 1.0, $CHCl_3$). GC: CP-Chirasil-Dex CB, column flow 1.50 mL/min, injector 250 °C; 130 °C, 175 °C (1 °C/min), 200 °C (5 °C/min), 10 min: (*R*)-**5**: t_R = 39.27 min (> 99%).

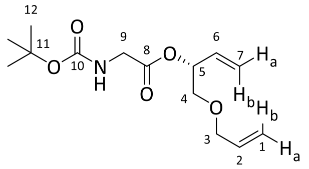

^{1}H NMR (400 MHz, $CDCl_3$): δ = 1.45 (s, 9 H, 12-H), 3.55 (m, 2 H, 4-H), 3.96 (d, $^3J_{9,NH}$ = 5.3 Hz, 2 H, 9-H), 4.01 (m, 2 H, 3-H), 5.00 (br s, 1 H, NH), 5.19 (ddt, $^3J_{1a,2}$ = 10.5 Hz, $^2J_{1a,1b}$ = 1.5 Hz, $^4J_{1a,3}$ = 1.3 Hz, 1 H, 1-H_a), 5.27 (m, 2 H, 1-H_b, 7-H_a), 5.34 (ddd, $^3J_{7b,6}$ = 17.3 Hz, $^2J_{7b,7a}$ ≈ $^4J_{7b,5}$ = 1.3 Hz, 1 H, 7-H_b), 5.50 (dt, $^3J_{5,6}$ = 6.0 Hz, $^3J_{5,4}$ = 5.0 Hz, 1 H, 5-H), 5.82 (ddd, $^3J_{6,7b}$ = 16.8 Hz, $^3J_{6,7a}$ = 10.5 Hz, $^3J_{6,5}$ = 6.3 Hz, 1 H, 6-H), 5.87 (ddt, $^3J_{2,1b}$ = 17.3 Hz, $^3J_{2,1a}$ = 10.3 Hz, $^3J_{2,3}$ = 5.5 Hz, 1 H, 2-H) ppm. ^{13}C NMR (100 MHz, $CDCl_3$): δ = 28.3 (q, C-12), 42.5 (t, C-9), 71.0 (t, C-4), 72.2 (t, C-3), 74.3 (d, C-5), 79.9 (s, C-11), 117.4 (t, C-1), 118.6 (t, C-7), 132.7 (d, C-6), 134.3 (d, C-2), 155.6 (s, C-10), 169.6 (s, C-8) ppm. HRMS (CI) calculated for $C_{14}H_{24}NO_5$ [M + H]$^+$: 286.1649, found: 286.1652.

(*S*,*E*)-6-(Allyloxy)-2-((*tert*-butoxycarbonyl)amino)hex-4-enoic acid (6)

Prepared in a manner similar to [1].

Diisopropylamine (1.00 mL, 710 mg, 7.02 mmol) was dissolved in 7.0 mL THF abs. and cooled to −78 °C before 4.00 mL (6.4 mmol) *n*-BuLi (1.6 M in hexanes) were added dropwise. The cooling bath was removed and the LDA solution was stirred at room temperature for 15 min.

A solution of 571 mg (2.00 mmol) **5** in 10.0 mL THF abs. was added to 330 mg (2.42 mmol) zinc chloride (dried *in high-vacuo*) and the mixture was cooled to −78 °C. The freshly prepared LDA solution was added slowly to the amino acid ester/zinc chloride solution at −78 °C. After complete addition, the remaining dry ice was removed from the cooling bath and the reaction was allowed to warm to room temperature overnight. For workup, the reaction mixture was

diluted with ether and 1 M $KHSO_4$ sol. was added. The layers were separated and the aqueous phase was extracted three times with ether. The combined organic layers were dried over Na_2SO_4 and concentrated *in vacuo*. 569 mg (1.99 mmol, er > 99:1, 99%) of acid **6** were obtained as a yellow oil. R_f (**6**) = 0.07 (silica gel, petroleum ether/ethyl acetate 60:40). $[\alpha]_D^{20}$ = +26.4 (*c* = 1.0, $CHCl_3$). GC: A small amount was derivatized using TMS-diazomethan for GC-analysis. CP-Chirasil-Dex CB, column flow 1.50 mL/min, injector 250 °C; 145 °C, 5 min, 180 °C (1 °C/min), 15 min, 200 °C (20 °C/min), 5 min: t_R = 28.95 min (> 99%).

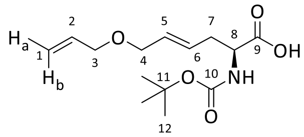

^{1}H NMR (400 MHz, $CDCl_3$): δ = 1.44 (s, 9 H, 12-H), 2.57 (m, 2 H, 7-H), 3.95 – 3.98 (m, 4 H, 3-H, 4-H), 4.39 (m, 1 H, 8-H), 5.05 (d, $^3J_{NH,8}$ = 7.1 Hz, 1 H, NH), 5.18 (d, $^3J_{1a,2}$ = 10.3 Hz, 1 H, 1-H_a), 5.27 (dd, $^3J_{1b,2}$ = 17.3 Hz, $^2J_{1b,1a}$ = 1.6 Hz, 1 H, 1-H_b), 5.59 – 5.74 (m, 2 H, 5-H, 6-H), 5.90 (ddt, $^3J_{2,1b}$ = 17.1 Hz, $^3J_{2,1a}$ = 10.4 Hz, $^3J_{2,3}$ = 5.8 Hz, 1 H, 2 H), 9.33 (br s, 1 H, COOH) ppm. ^{13}C NMR (100 MHz, $CDCl_3$): δ = 28.3 (q, C-12), 34.9 (t, C-7), 52.8 (d, C-8), 70.2 (t, C-3 /C-4), 71.0 (d, C-3/C-4), 80.4 (s, C-11), 117.3 (t, C-1), 127.3 (t, C-6), 131.1 (d, C-7), 134.5 (d, C-2), 155.6 (s, C-10), 175.7 (s, C-9) ppm. HRMS (CI) calculated for $C_{14}H_{24}NO_5$ $[M + H]^+$: 286.1649, found: 286.1654.

(*R*)-1-(Allyloxy)but-3-en-2-yl glycinate hydrochloride (7)

To a solution of 2.1 mL (1.66 g, 51.8 mmol) methanol in 8.8 mL ethyl acetate were added 3.6 mL (3.96 g, 50.4 mmol) acetyl chloride at 0 °C and the reaction mixture was stirred for 30 min. The resulting HCl sol. was then transferred to 1.47 g (5.16 mmol) **5** at room temperature and the reaction mixture was stirred for 1 h. The reaction mixture was concentrated *in vacuo* and the crude product was dried by lyophilization. 1.10 g (4.96 mmol, 96%) of hydrochloride salt **7** were obtained as an off-white solid, mp.: 53 °C. $[\alpha]_D^{20}$ = −6.5 (*c* = 1.0, $CHCl_3$).

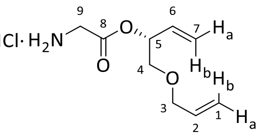

^{1}H NMR (400 MHz, $CDCl_3$): δ = 3.56 (d, $^3J_{4,5}$ = 5.3 Hz, 2 H, 4-H), 4.00 (m, 4 H, 3-H, 9-H), 5.18 (dd, $^3J_{7a,6}$ = 10.3 Hz, $^2J_{7a,7b}$ = 1.3 Hz, 1 H, 7-H_a), 5.26 (m, 2 H, 1-H_a, 1-H_b), 5.39 (d, $^3J_{7b,6}$ = 17.3 Hz, 1 H, 7-H_b), 5.52 (dt, $^3J_{5,6}$ = 5.5 Hz, $^3J_{5,4}$ = 5.5 Hz, 1 H, 5-H), 5.82 (ddd, $^3J_{6,7b}$ = 17.1 Hz, $^3J_{6,7a}$ = 10.8 Hz, $^3J_{6,5}$ = 6.3 Hz, 1 H, 6-H), 5.86 (ddt, $^3J_{2,1b}$ = 17.3 Hz, $^3J_{2,1a}$ = 10.8 Hz, $^3J_{2,3}$ = 5.5 Hz, 1 H, 2-H), 8.56 (br s, 3 H, NH_3) ppm. ^{13}C NMR (100 MHz, $CDCl_3$): δ = 40.7 (t, C-9), 70.8 (t, C-4), 72.3 (t, C-3), 75.7 (d, C-5), 117.6 (t, C-1), 119.2 (t, C-7), 132.3 (d, C-6), 134.3 (d, C-2), 167.1 (s, C-8) ppm. HRMS (CI) calculated for $C_9H_{16}NO_3$ $[M + H]^+$: 186.1125, found: 186.1130.

(*R*)-1-(Allyloxy)but-3-en-2-yl ((*R*)-2-(((benzyloxy)carbonyl)amino)-3-(4-methoxyphenyl)-propanoyl)-L-isoleucyl-L-prolylglycinate (8a)

Cbz-MeOTyr-Ala-Pro-OH (888 mg, 1.65 mmol) and 332 mg (1.50 mmol) **7** were dissolved in 15.0 mL MeCN abs. before 528 mg (1.64 mmol) TBTU, 249 mg (1.63 mmol) HOBt and 588 µL (435 mg, 3.37 mmol) diisopropylethylamine were added subsequently at room temperature. The reaction mixture was stirred at room temperature overnight and concentrated *in vacuo*. The residue was dissolved in dichloromethane and 1 M HCl sol. was added. The layers were separated and the aqueous phase was extracted three times with dichloromethane. The combined organic layers were washed with brine, sat. $NaHCO_3$ sol., and dried over Na_2SO_4. After purification by column chromatography (silica gel, petroleum ether/ethyl acetate 100:0, 80:20, 50:50), 727 mg (1.03 mmol, dr ≈ 90:10, 69%) of tetrapeptide **8a** were obtained as an off-white solid, mp.: 47–48 °C. R_f (**8a**) = 0.04 (silica gel, petroleum ether/ethyl acetate 50:50). $[\alpha]_D^{20}$ = −64.7 (*c* = 0.5, $CHCl_3$).

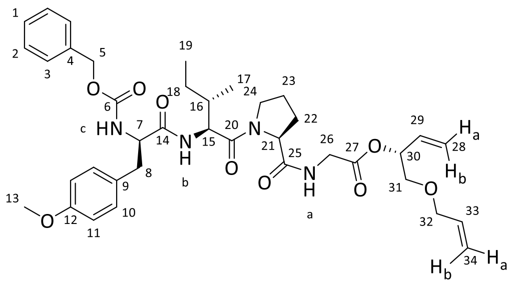

Major diastereomer: ^{1}H NMR (400 MHz, $CDCl_3$): δ = 0.61 – 0.72 (m, 4 H, 18-H_a, 19-H), 0.80 – 0.97 (m, 1 H, 18-H_b), 0.88 (d, $^3J_{13,12}$ = 6.5 Hz, 3 H, 17-H), 1.57 (m, 1 H, 16-H), 1.92 – 2.07 (m, 2 H, 22-H_a, 23-H_a), 2.13 (m, 1 H, 23-H_b), 2.24 (m, 1 H, 22-H_b), 2.93 (m, 2 H, 8-H), 3.52 (d, $^3J_{31,30}$ = 5.3 Hz, 2 H, 31-H), 3.65 – 3.81 (m, 2 H, 24-H_a, 26-H_a), 3.74 (s, 3 H, 13-H), 3.87 (dt, $^2J_{24b,24a}$ = 9.5 Hz, $^3J_{24b,23}$ = 7.5 Hz, 1 H, 24-H_b), 3.98 (m, 2 H, 32-H), 4.18 (dd, $^2J_{26b,26a}$ = 18.4 Hz, $^3J_{26b,NH}$ = 5.6 Hz, 1 H, 26-H_b), 4.50 (dd, $^3J_{15,NH}$ ≈ $^3J_{15,16}$ = 9.0 Hz, 1 H, 15-H), 4.66 – 4.76 (m, 2 H, 7-H, 21-H), 5.08 (s, 2 H, 5-H), 5.17 (ddd, $^3J_{34a,33}$ = 10.5 Hz, $^2J_{34a,34b}$ ≈ $^4J_{34a,32}$ = 1.3 Hz, 1 H, 34-H_a), 5.20 – 5.33 (m, 3 H, 28-H, 34-H_b), 5.44 (dt, $^3J_{30,29}$ ≈ $^3J_{30,31}$ = 5.7 Hz, 1 H, 30-H), 5.48 (m, 1 H, NH_c), 5.74 – 5.90 (m, 2 H, 29-H, 33-H), 6.76 (d, $^3J_{11,10}$ = 8.8 Hz, 2 H, 11-H), 7.05 (d, $^3J_{10,11}$ = 8.3 Hz, 2 H, 10-H), 7.27 – 7.36 (m, 6 H, 1-H, 2-H, 3-H, NH_b), 7.67 (br s, 1 H, NH_a) ppm. ^{13}C NMR (100 MHz, $CDCl_3$): δ = 10.9 (q, C-19), 15.2 (q, C-17), 23.9 (t, C-18), 24.7 (t, C-23), 28.4 (t, C-22), 37.4 (d, C-16), 38.8 (t, C-8), 41.4 (t, C-26), 47.9 (t, C-24), 54.8 (d, C-15), 55.1 (q, C-13), 55.5 (d, C-7), 59.6 (d, C-21), 67.0 (t, C-5), 71.0 (t, C-31), 72.2 (t, C-32), 74.3 (d, C-30), 113.9 (d, C-11), 117.4 (t, C-34), 118.5 (t, C-28), 128.0, 128.1, 128.2 (3 d, C-1/C-3/C-9), 128.5 (d, C-2), 130.2 (d, C-10), 132.8 (d, C-29), 134.3 (d, C-33), 136.2 (s, C-4), 155.7 (s, C-6), 158.5 (s, C-12), 168.8 (s, C-14/C-20/C-27) ppm. 3 signals of C-14/C-20/C-25/C-27 weren't observed in the ^{13}C NMR spectrum. Minor diastereomer (selected signals): ^{1}H NMR (400 MHz, $CDCl_3$): δ = 0.81 (d, $^3J_{17,16}$ = 6.8 Hz, 3 H, 17-H), 6.80 (d, $^3J_{11,10}$ = 8.3 Hz, 2 H, 11-H) ppm. HRMS (CI) calculated for $C_{38}H_{51}N_4O_9$ $[M + H]^+$: 707.3651, found: 707.3642.

(*R*)-1-(Allyloxy)but-3-en-2-yl ((*R*)-2-((*tert*-butoxycarbonyl)amino)-3-(4-methoxyphenyl)-propanoyl)-L-isoleucyl-L-prolylglycinate (8b)

To a solution of 1.19 g (2.35 mmol) Boc-MeOTyr-Ala-Pro-OH and 525 mg (2.37 mmol) **7** in 19 mL acetonitrile were added 757 mg (2.36 mmol) TBTU. Afterwards, 0.84 mL (622 mg, 4.81 mmol) diisopropylethylamine were added dropwise and the reaction mixture was stirred at room temperature for 5 h. For workup, the reaction mixture was concentrated *in vacuo* and

the residue was dissolved in dichloromethane. 1 M HCl sol. was added and the layers were separated. The aqueous phase was extracted three times with dichloromethane, the combined organic phases were washed with sat. $NaHCO_3$ sol. and dried over Na_2SO_4. The crude product was purified by column chromatography (silica gel, petroleum ether/ethyl acetate 100:0, 80:20, 50:50) to give 1.24 g (1.84 mmol, dr > 99:1, 78%) of tetrapeptide **8b** as an off-white solid, mp.: 53 °C. R_f (**8b**) = 0.08 (silica gel, petroleum ether/ethyl acetate 50:50). $[\alpha]_D^{20}$ = −61.2 (*c* = 1.0, $CHCl_3$). HPLC: Reprosil, *n*-hexane/iPrOH 70:30, 1 mL/min, 20 °C: t_R = 27.7 min (> 99%). LCMS: Luna, MeCN/H_2O + 0.1% HCOOH 50:50, gradient 90:10, 0.6 mL/min, t_R = 9.67 min ($[M + H]^+$ = 673).

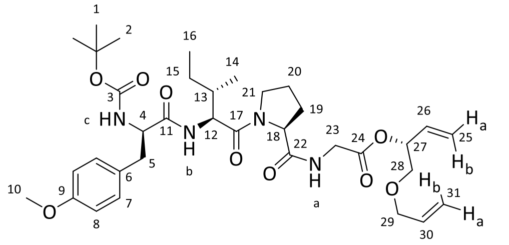

^{1}H NMR (400 MHz, $CDCl_3$): δ = 0.71 (m, 3 H, 16-H), 0.80 (m, 1 H, 15-H_a), 0.89 (d, $^3J_{14,13}$ = 6.4 Hz, 3 H, 14-H), 1.06 (m, 1 H, 15-H_b), 1.40 (s, 9 H, 1-H), 1.62 (m, 1H, 13-H), 1.99 (m, 2 H, 19-H_a, 20-H_a), 2.13 (m, 1 H, 20-H_b), 2.25 (m, 1 H, 19-H_b), 2.93 (m, 2 H, 5-H), 3.51 (d, $^3J_{28,27}$ = 5.5 Hz, 2 H, 28-H), 3.68 (m, 1 H, 21-H_a), 3.75 (s, 3 H, 10-H), 3.84 (m, 1 H, 23-H_a), 3.88 (m, 1 H, 21-H_b), 3.98 (m, 2 H, 29-H), 4.16 (dd, $^2J_{23b,23a}$ = 18.3 Hz, $^3J_{23b,NH}$ = 5.5 Hz, 1 H, 23-H_b), 4.51 (dd, $^3J_{12,13} \approx {}^3J_{12,NH}$ = 8.8 Hz, 1 H, 12-H), 4.63 (m, 1 H, 4-H), 4.68 (m, 1 H, 18-H), 5.14 (m, 1 H, NH_c), 5.22 (m, 4 H, 25-H, 31-H), 5.43 (dt, $^3J_{27,26}$ = 5.8 Hz, $^3J_{27,28}$ = 5.5 Hz, 1 H, 27-H), 5.82 (m, 2 H, 26-H, 30-H), 6.78 (d, $^3J_{8,7}$ = 8.5 Hz, 2 H, 8-H), 7.07 (d, $^3J_{7,8}$ = 8.5 Hz, 2 H, 7-H), 7.21 (m, 1 H, NH_b), 7.66 (m, 1 H, NH_a) ppm. ^{13}C NMR (100 MHz, $CDCl_3$): δ = 10.9 (q, C-16), 15.2 (q, C-14), 24.1 (t, C-15), 24.8 (t, C-20), 27.7 (t, C-19), 28.3 (q, C-1), 37.5 (d, C-13), 38.4 (t, C-5), 41.3 (t, C-23), 47.8 (t, C-21), 54.8 (d, C-12), 55.1 (q, C-10), 55.2 (d, C-4), 59.6 (d, C-18), 71.0 (t, C-28), 72.2 (t, C-29), 74.3 (d, C-27), 80.1 (s, C-2), 113.9 (d, C-8), 117.4, 118.5 (2 t, C-25/C-31), 128.3 (s, C-6), 130.2 (t, C-7), 132.7, 134.2 (2 d, C-26/C-30), 155.2 (s, C-3), 158.5 (s, C-9), 168.8 (s, C-24), 171.1 (s, C-17/C-22), 171.5 (s, C-11), 171.8 (s, C-17/C-22) ppm. HRMS (CI) calculated for $C_{35}H_{53}N_4O_9$ $[M+H]^+$: 673.38078, found: 673.3814.

(*S*,*E*)-6-(Allyloxy)-2-((*S*)-1-(((*R*)-2-((*tert*-butoxycarbonyl)amino)-3-(4-methoxyphenyl)-pro-panoyl)-L-isoleucyl)pyrrolidine-2-carboxamido)hex-4-enoic acid (9b)

Prepared in a manner similar to [1].

A solution of 0.91 mL (646 mg, 6.38 mmol) diisopropylamine in 6.5 mL THF abs. was cooled to −78 °C before 3.6 mL (5.76 mmol) *n*-BuLi (1.6 M in hexanes) were added dropwise. The cooling bath was removed and the reaction mixture was stirred at room temperature for 15 min.

435 mg (3.19 mmol) zinc chloride (dried *in high-vacuo*) was treated with a solution of 713 mg (1.06 mmol) **8b** in 13 mL THF abs. at room temperature. After cooling to −78 °C, the previously prepared LDA solution was added dropwise to the zinc chloride/tetrapeptide ester solution. Upon complete addition, the remaining dry ice was removed from the cooling bath and the reaction was allowed to warm to room temperature overnight. For workup, the reaction mixture was diluted with ether and 1 M $KHSO_4$ sol. was added. The layers were separated and the aqueous phase was extracted three times with diethyl ether. The combined organic layers were dried over Na_2SO_4 and concentrated *in vacuo*. After lyophilization, 713 mg (1.06 mmol, dr > 99:1, quant.) of acid **9b** were obtained as a slightly yellow solid, mp.: 67–68 °C. R_f (**9b**) =

0.02 (silica gel, petroleum ether/ethyl acetate 50:50 + 1% HOAc). $[\alpha]_D^{20}$ = −33.3 (*c* = 1.0, $CHCl_3$). HPLC: A small sample was derivatized with TMS-diazomethane for HPLC analysis. Reprosil, *n*-hexane/iPrOH 70:30, 1 mL/min, 20 °C: t_R = 32.3 min (> 99%). LCMS: Luna, $MeCN/H_2O$ + 0.1% HCOOH 50:50, gradient 90:10, 0.6 mL/min, t_R = 17.6 min ($[M + Na]^+$ = 695).

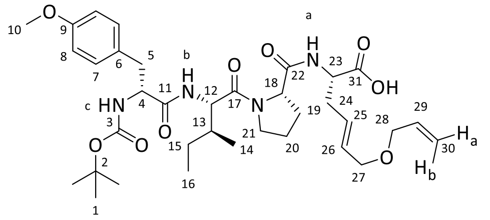

^{1}H NMR (400 MHz, $CDCl_3$): δ = 0.79 (t, $^3J_{16,15}$ = 7.4 Hz, 3 H, 16-H), 0.88 (d, $^3J_{14,13}$ = 6.5 Hz, 3 H, 14-H), 0.98 (m, 1 H, 15-H_a), 1.37 (s, 9 H, 1-H), 1.38 (m, 1 H, 15-H_b), 1.69 (m, 1 H, 13-H), 1.89 – 2.20 (m, 3 H, 19-H_a, 20-H), 2.40 (m, 1 H, 19-H_b), 2.49 (ddd, $^2J_{24a,24b}$ = 13.6 Hz, $^3J_{24a,23}$ ≈ $^3J_{24a,25}$ = 5.9 Hz, 1 H, 24-H_a), 2.61 (ddd, $^2J_{24b,24a}$ = 13.9 Hz, $^3J_{24b,23}$ ≈ $^3J_{24b,25}$ = 5.0 Hz, 1 H, 24-H_b), 2.96 (m, 2 H, 5-H), 3.64 (m, 1 H, 21-H_a), 3.76 (s, 3 H, 10-H), 3.86 (d, $^3J_{27,26}$ = 3.9 Hz, 2 H, 27-H), 3.91 (d, $^3J_{28,29}$ = 5.6 Hz, 2 H, 28-H), 3.94 (m, 1 H, 21-H_b), 4.40 (m, 1 H, 4-H), 4.49 (m, 1 H, 12-H), 4.53 (td, $^3J_{23,24}$ ≈ $^3J_{23,NH}$ = 6.4 Hz, 1 H, 23-H), 4.65 (d, $^3J_{18,19a/b}$ = 6.5 Hz, 1 H, 18-H), 5.15 (d, $^3J_{30a,29}$ = 10.2 Hz, 1 H, 30-H_a), 5.16 (m, 1 H, NH_c), 5.23 (dd, $^3J_{30b,29}$ = 17.2 Hz, $^2J_{30b,30a}$ = 1.6 Hz, 1 H, 30-H_b), 5.54 – 5.67 (m, 2 H, 25-H, 26-H), 5.86 (ddd, $^3J_{29,30b}$ = 16.0 Hz, $^3J_{29,30a}$ = 11.0 Hz, $^3J_{29,28}$ = 5.6 Hz, 1 H, 29-H), 6.79 (d, $^3J_{8,7}$ = 8.6 Hz, 2 H, 8-H), 7.07 (d, $^3J_{7,8}$ = 8.4 Hz, 2 H, 7-H), 7.52 (d, $^3J_{NH,12}$ = 8.4 Hz, 1 H, NH_b), 7.60 (m, 1 H, NH_a) ppm. The signal of C-13 wasn't observed in the ^{1}H NMR spectrum. ^{13}C NMR (100 MHz, $CDCl_3$): δ = 10.7 (q, C-16), 15.1 (q, C-14), 24.6 (t, C-15), 24.8 (t, C-20), 27.7 (t, C-19), 28.2 (q, C-1), 35.3 (t, C-24), 37.0 (d, C-13), 38.0 (t, C-5), 48.2 (t, C-21), 52.6 (d, C-23), 55.0 (d, C-12), 55.2 (q, C-10), 55.6 (d, C-4), 60.5 (d, C-18), 70.3 (t, C-27), 71.1 (t, C-28), 79.8 (s, C-2), 113.9 (d, C-8), 117.1 (t, C-30), 127.8 (d, C-25/C-26), 128.4 (s, C-6), 130.3 (d, C-7), 130.5 (d, C-25/C-26), 134.6 (d, C-29), 155.1 (s, C-3), 158.5 (s, C-9), 170.3 (s, C-22), 172.0 (s, C-11), 173.2 (s, C-31) ppm. The signal of C-17 wasn't observed in the ^{13}C NMR spectrum. HRMS (CI) calculated for $C_{35}H_{53}N_4O_9$ $[M + H]^+$: 673.3807, found: 673.3816.

Pentafluorophenyl (*S*,*E*)-6-(allyloxy)-2-((*S*)-1-(((*R*)-2-((*tert*-butoxycarbonyl)amino)-3-(4-methoxyphenyl)propanoyl)-L-isoleucyl)pyrrolidine-2-carboxamido)hex-4-enoate (10)

To a solution of 484 mg (0.705 mmol) **9b** in 7.0 mL dichloromethane abs. were added 149 mg (0.809 mmol) pentafluorophenol and 149 mg (0.777 mmol) EDC·HCl at 0 °C. The reaction mixture was allowed to warm to room temperature overnight. The reaction mixture was diluted with dichloromethane and 1 M HCl sol. was added. The layers were separated and the aqueous phase was extracted three times with dichloromethane. The combined organic phases were washed with sat. $NaHCO_3$ sol. and dried over Na_2SO_4. 573 mg (0.683 mmol, dr ≈ 96:4, 97%) of pentafluorophenyl ester **10** were obtained after lyophilisation, mp.: 52–53 °C. R_f (**10**) = 0.16 (silica gel, petroleum ether/ethyl acetate 50:50). $[\alpha]_D^{20}$ = −43.1 (*c* = 1.0, $CHCl_3$). LCMS: Luna, $MeCN/H_2O$ + 0.1% HCOOH 10:90, gradient 90:10, 0.6 mL/min, t_R = 23.0 min ($[M + H]^+$ = 839).

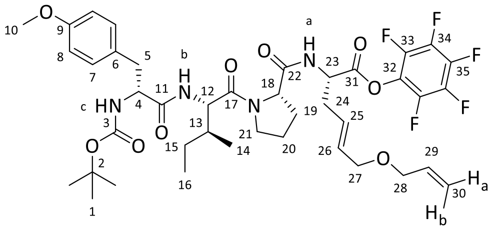

^{1}H NMR (500 MHz, 373 K, DMSO-d_6): δ = 0.81 (t, $^3J_{16,15}$ = 7.0 Hz, 3 H, 16-H), 0.88 (m, 1 H, 15-H$_a$), 0.89 (d, $^3J_{14,13}$ = 6.7 Hz, 3 H, 14-H), 1.05 (m, 1 H, 15-H$_b$), 1.34 (s, 9 H, 1-H), 1.44 (m, 1 H, 13-H), 1.73 – 2.06 (m, 5 H, 19-H, 20-H, 24-H$_a$), 2.40 (m, 1 H, 24-H$_b$), 2.73 (dd, $^2J_{5a,5b}$ = 13.9 Hz, $^3J_{5a,4}$ = 9.1 Hz, 1 H, 5-H$_a$), 2.91 (dd, $^2J_{5b,5a}$ = 13.9 Hz, $^3J_{5b,4}$ = 5.4 Hz, 1 H, 5-H$_b$), 3.57 (m, 1 H, 21-H$_a$), 3.74 (s, 3 H, 10-H), 3.88 – 3.94 (m, 5 H, 27-H, 28-H, 21-H$_b$), 4.20 (m, 1 H, 4-H), 4.31 (m, 1 H, 23-H), 4.41 (t, $^3J_{18,19}$ = 8.2 Hz, 1 H, 18-H), 4.47 (m, 1 H, 23-H), 5.13 (dd, $^3J_{30a,29}$ = 10.4 Hz, $^2J_{30a,30b}$ = 1.4 Hz, 1 H, 30-H$_a$), 5.24 (d, $^3J_{30b,29}$ = 17.4 Hz, 1 H, 30-H$_b$), 5.59 – 5.70 (m, 2 H, 25-H, 26-H), 5.89 (dddd, $^3J_{29,30b}$ = 15.9 Hz, $^3J_{29,30a}$ = 10.7 Hz, $^3J_{29,28a}$ = 5.4 Hz, $^3J_{29,28b}$ = 2.1 Hz, 1 H, 29-H), 6.38 (br s, 1 H, NH$_c$), 6.81 (d, $^3J_{8,7}$ = 8.6 Hz, 2 H, 8-H), 7.14 (d, $^3J_{7,8}$ = 8.4 Hz, 2 H, 7-H), 7.56 – 7.67 (m, 2 H, NH$_a$, NH$_b$) ppm. ^{13}C NMR (100 MHz, $CDCl_3$): δ = 10.9 (q, C-16), 15.2 (q, C-14), 24.2 (t, C-15), 24.8 (t, C-20), 27.7 (t, C-19), 28.2 (q, C-1), 34.5 (t, C-24), 37.5 (d, C-13), 38.2 (t, C-5), 47.9 (t, C-21), 52.2 (d, C-23), 54.8 (d, C-4/C-12), 55.1 (q, C-10), 55.2 (d, C-4/C-12), 59.5 (d, C-18), 70.0 (t, C-27), 71.1 (t, C-28), 114.0 (d, C-8), 117.0 (t, C-30), 125.5 (d, C-25), 128.3 (s, C-6), 130.2 (d, C-7), 132.2 (d, C-26), 134.6 (d, C-29), 155.1 (s, C-3), 158.6 (s, C-9), 167.6 (s, C-31), 171.2, 171.4, 171.9 (3 s, C-11/C-17/C-22) ppm. The signals of C-2, C-32, C-33, C-34, and C-35 weren't observed in the ^{13}C NMR spectrum. Minor diastereomer (selected signals): ^{1}H NMR (500 MHz, 373 K, DMSO-d_6): δ = 4.72 (m, 1 H, 23-H), 7.05 (d, $^3J_{8,7}$ = 8.5 Hz, 2 H, 8-H), 7.26 (d, $^3J_{7,8}$ = 8.7 Hz, 2 H, 7-H). ^{13}C NMR (100 MHz, $CDCl_3$): δ = 10.8 (q, C-16), 15.0 (q, C-14), 34.7 (t, C-24), 52.3 (d, C-23), 114.0 (d, C-8), 125.5 (d, C-25), 132.3 (d, C-26), 134.6 (d, C-29). HRMS (CI) calculated for $C_{41}H_{52}F_5N_4O_9$ [M + H]$^+$: 839.3649, found: 839.3666.

(3*S*,6*R*,9*S*,14a*S*)-3-((*E*)-4-(Allyloxy)but-2-en-1-yl)-9-((*S*)-*sec*-butyl)-6-(4-methoxybenzyl)-decahydropyrrolo[1,2-*a*][1,4,7,10]tetraazacyclododecine-1,4,7,10-tetraone (11)

A solution of 90.6 mg (0.108 mmol) **10** in 0.3 mL dioxane was treated with 0.54 mL (2.16 mmol) HCl (4 M in dioxane) at room temperature. The reaction mixture was stirred for 1 h at room temperature and concentrated *in vacuo*. The residue was redissolved in 25 mL chloroform and added dropwise to a vigorously stirred emulsion of 50 mL chloroform in 50 mL sat. $NaHCO_3$ at room temperature. The reaction mixture was stirred for 4 h. The layers were then separated and the aqueous phase was extracted two times with dichloromethane. The combined organic phases were dried over Na_2SO_4 and concentrated *in vacuo*. Purification of the crude product by column chromatography (silica gel, dichloromethane/MeOH 100:0, gradient 95:5) afforded 29.0 mg (52.3 µmol, dr 87:13, 48% over two steps) of cyclic peptide **11** as an off-white solid, mp.: 250 °C (decomposition). A small sample was further purified by reversed-phase column chromatography (silica gel C-18, MeCN/H_2O 10:90, gradient 90:10) to partially separate the epimers and for analytical purposes. R_f (**11**) = 0.30 (silica gel, dichloromethane/MeOH 95:5). $[\alpha]_D^{20}$ = −76.6 (*c* = 1.0, $CHCl_3$). LCMS: Luna, MeCN/H_2O + 0.1% HCOOH 10:90, gradient 90:10, 0.6 mL/min, t_{R1} = 16.4 min (13%, [M + H]$^+$ = 555), t_{R2} = 17.4 min (87%, [M + H]$^+$ = 555).

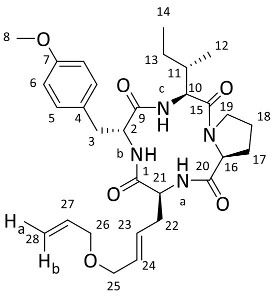

^{1}H NMR (500 MHz, $CDCl_3$): δ = 0.71 (t, $^3J_{14,13}$ = 7.4 Hz, 3 H, 14-H), 0.80 (d, $^3J_{12,11}$ = 6.3 Hz, 3 H, 12-H), 0.81 (m, 1 H, 13-H_a), 1.05 (m, 1 H, 13-H_b), 1.82 – 1.92 (m, 2 H, 11-H, 18-H_a), 1.96 (m, 1 H, 18-H_b), 2.14 (dd, $^2J_{17a,17b}$ = 12.8 Hz, $^3J_{17a,16}$ = 6.8 Hz, 1 H, 17-H_a), 2.23 (m, 1 H, 17-H_b), 2.49 (dt, $^2J_{22a,22b}$ = 13.9 Hz, $^3J_{22a,21}$ ≈ $^3J_{22a,23}$ = 7.3 Hz, 1 H, 22-H_a), 2.60 (dt, $^2J_{22b,22a}$ = 13.3 Hz, $^3J_{22b,21}$ ≈ $^3J_{22b,23}$ = 6.7 Hz, 1 H, 22-H_b), 2.84 (dd, $^2J_{3a,3b}$ = 13.6 Hz, $^3J_{3a,2}$ = 5.7 Hz, 1 H, 3-H_a), 3.19 (dd, $^2J_{3b,3a}$ = 13.6 Hz, $^3J_{3b,2}$ = 10.1 Hz, 1 H, 3-H_b), 3.61 (m, 1 H, 19-H_a), 3.69 (s, 3 H, 8-H), 3.76 (m, 1 H, 19-H_b), 3.98 (d, $^3J_{25,24}$ = 4.7 Hz, 2 H, 25-H), 3.93 (ddd, $^3J_{26,27}$ = 5.7 Hz, $^4J_{26,28a}$ ≈ $^4J_{26,28b}$ = 1.3 Hz, 2 H, 26-H), 4.52 (dd, $^3J_{10,11}$ ≈ $^3J_{10,NH}$ = 9.8 Hz, 1 H, 10-H), 4.63 (ddd, $^3J_{2,3b}$ = 10.4 Hz, $^3J_{2,3a}$ ≈ $^3J_{2,NH}$ = 5.7 Hz, 1 H, 2-H), 4.67 (m, 1 H, 21-H), 4.95 (d, $^3J_{16,17a/b}$ = 8.8 Hz, 1 H, 16-H), 5.17 (ddt, $^3J_{28a,29}$ = 10.4 Hz, $^2J_{28a,28b}$ = 1.6 Hz, $^4J_{28a,26}$ = 1.3 Hz, 1 H, 28-H_a), 5.25 (ddt, $^3J_{28b,29}$ = 17.3 Hz, $^2J_{28b,28a}$ ≈ $^4J_{28b,26}$ = 1.6 Hz, 1 H, 28-H_b), 5.56 – 5.66 (m, 2 H, 23-H, 24-H), 5.88 (ddd, $^3J_{27,28b}$ = 16.6 Hz, $^3J_{27,28a}$ = 10.6 Hz, $^3J_{27,26}$ = 5.5 Hz, 1 H, 29-H), 6.22 (br s, 1 H, NH_a), 6.57 (br s, 1 H, NH_b), 6.65 (d, $^3J_{6,5}$ = 8.5 Hz, 2 H, 6-H), 6.86 (br s, 1 H, NH_c), 7.00 (d, $^3J_{5,6}$ = 8.5 Hz, 2 H, 5-H) ppm. ^{13}C NMR (125 MHz, $CDCl_3$): δ = 11.3 (q, C-14), 15.9 (q, C-12), 21.8 (t, C-18), 24.1 (t, C-13), 31.1 (t, C-22), 31.6 (t, C-17), 34.6 (t, C-3), 36.3 (d, C-11), 47.0 (t, C-19), 53.3 (d, C-21), 55.0 (q, C-8), 55.8 (d, C-10), 57.4 (d, C-2), 61.0 (d, C-16), 70.3 (t, C-25), 71.0 (t, C-26), 113.7 (d, C-6), 117.0 (t, C-28), 127.9 (d, C-23), 128.6 (s, C-4), 130.3 (d, C-24), 130.5 (d, C-5), 134.7 (d, C-27), 158.3 (s, C-7), 170.2 (s, C-9), 170.6 (s, C-15), 173.5 (s, C-1), 174.5 (s, C-20) ppm. HRMS (CI) calculated for $C_{30}H_{43}N_4O_6$ $[M + H]^+$: 555.3177, found: 555.3169.

(3*S*,6*R*,9*S*,14a*S*)-3-Allyl-9-((*S*)-*sec*-butyl)-6-(4-methoxybenzyl)decahydropyrrolo[1,2-*a*]-[1,4,7,10]tetraazacyclododecine-1,4,7,10-tetraone (12)

26.5 mg (48.5 µ mol) **11** and 2.0 mg (2.39 µmol) Grubbs I catalyst were dissolved in 2.0 mL dichloromethane abs. and heated to 45 °C in a sealed tube for 20 h. For workup, 8.5 µL (9.4 mg, 120 µmol) DMSO were added and stirring was continued for 24 h. The mixture was concentrated *in vacuo* and purified by column chromatography (silica gel, dichloromethane/MeOH 100:0, gradient 95:5) to yield 13.0 mg (26.8 µmol, 55%) of peptide **12** as an off-white solid, mp.: 280 °C (decomposition). R_f (**12**) = 0.40 (silica gel, dichloromethane/MeOH 95:5). LCMS: Luna, MeCN/H_2O + 0.1% HCOOH 10:90, gradient 90:10, 0.8 mL/min, t_R = 10.1 min ($[M + H]^+$ = 485).

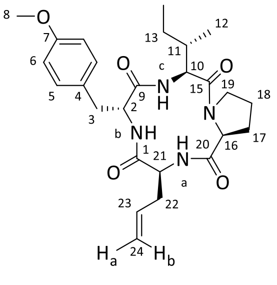

^{1}H NMR (500 MHz, 373 K, tetrachloroethane-d_2): δ = 0.83 (t, $^{3}J_{14,13}$ = 7.4 Hz, 3 H, 14-H), 0.88 (d, $^{3}J_{12,11}$ = 6.6 Hz, 3 H, 12-H), 0.96 (m, 1 H, 13-H$_a$), 1.27 (m, 1 H, 13-H$_b$), 1.87 – 1.98 (m, 2 H, 11-H, 18-H$_a$), 2.01 (m, 1 H, 18-H$_b$), 2.18 (m, 1 H, 17-H$_a$), 2.28 (m, 1 H, 17-H$_b$), 2.48 (dt, $^{2}J_{22a,22b}$ = 14.8 Hz, $^{3}J_{22a,21}$ ≈ $^{3}J_{22a,23}$ = 7.4 Hz, 1 H, 22-H$_a$), 2.62 (dt, $^{2}J_{22b,22a}$ = 14.5 Hz, $^{3}J_{22b,21}$ ≈ $^{3}J_{22b,23}$ = 6.7 Hz, 1 H, 22-H$_b$), 2.88 (dd, $^{2}J_{3a,3b}$ = 14.1 Hz, $^{3}J_{3a,2}$ = 6.3 Hz, 1 H, 3-H$_a$), 3.25 (dd, $^{2}J_{3b,3a}$ = 14.1 Hz, $^{3}J_{3b,2}$ = 9.1 Hz, 1 H, 3-H$_b$), 3.68 (m, 2 H, 19-H), 3.81 (s, 3 H, 8-H), 4.54 (dd, $^{3}J_{10,11}$ ≈ $^{3}J_{10,NH}$ = 9.3 Hz, 1 H, 10-H), 4.54 (m, 1 H, 21-H), 4.63 (m, 1 H, 2-H), 4.68 (d, $^{3}J_{16,17a/b}$ = 8.8 Hz, 1 H, 16-H), 5.18 (m, 2 H, 24-H), 5.67 (d, $^{3}J_{NH,21}$ = 10.0 Hz, 1 H, NH$_a$), 5.82 (m, 1 H, 23-H), 5.87 (d, $^{3}J_{NH,10}$ = 10.0 Hz, 1 H, NH$_c$), 6.29 (d, $^{3}J_{NH,2}$ = 10.7 Hz, 1 H, NH$_b$), 6.84 (d, $^{3}J_{6,5}$ = 8.8 Hz, 2 H, 6-H), 7.16 (d, $^{3}J_{5,6}$ = 8.8 Hz, 2 H, 5-H) ppm. ^{13}C NMR (125 MHz, 373 K, tetrachloroethane-d_2): δ = 11.0 (q, C-14), 15.7 (q, C-12), 21.6 (t, C-18), 24.2 (t, C-13), 31.2 (t, C-17), 32.2 (t, C-22), 34.2 (t, C-3), 36.6 (d, C-11), 46.9 (t, C-19), 52.9 (d, C-21), 55.2 (q, C-8), 55.5 (d, C-10), 56.3 (d, C-2), 61.2 (d, C-16), 114.1 (d, C-6), 118.2 (t, C-24), 128.8 (s, C-4), 130.0 (d, C-5), 132.5 (d, C-23), 158.5 (s, C-7), 170.0 (s, C-9), 170.1 (s, C-15), 173.0 (s, C-1), 174.4 (s, C-20). HRMS (CI) calculated for $C_{26}H_{36}N_4O_5$ $[M]^+$: 484.2686, found: 484.2684.

Methyl 4-((3*S*,6*R*,9*S*,14a*S*)-9-((*S*)-*sec*-butyl)-6-(4-methoxybenzyl)-1,4,7,10-tetraoxotetradecahydropyrrolo[1,2-*a*][1,4,7,10]tetraazacyclododecin-3-yl)butanoate (13)

To a solution of 14.7 mg (26.5 µmol) **11** in 0.53 mL dichloromethane abs. were added 6.44 µL pyridine and the reaction mixture was cooled to −78 °C. The reaction mixture was then treated with ozone until the mixture turned blue. The dissolved ozone was removed with oxygen and the reaction mixture was allowed to warm to room temperature within 15 min. Afterwards, a solution of 35.4 mg (106 µmol) methyl 2-(triphenyl-λ^5-phosphaneylidene)acetate in 0.53 mL dichloromethane abs. was added and stirring was continued for 3 h. For workup, the reaction mixture was diluted with ether and 1 M HCl sol. was added. The layers were separated and the aqueous phase was extracted three times with diethyl ether. The combined organic layers were washed with sat. $NaHCO_3$ and dried over Na_2SO_4. The crude product was purified by column chromatography (silica gel, dichloromethane/MeOH 100:0, gradient 95:5) to give 6.8 mg (8.36 µmol, *E*/*Z* = 3:1, 32%) of α,β-unsaturated ester, which contained triphenylphosphine oxide as impurity.

The ester (6.6 mg, 8.15 µmol) was then dissolved in 1.0 mL MeOH at room temperature before 2.0 mg palladium on charcoal (10 wt % Pd) were added and hydrogenated for 6.5 h. After filtration through a pad of Celite® the filtrate was concentrated *in vacuo*, and the crude product was purified by column chromatography (silica gel, dichloromethane/MeOH 100:0, gradient 95:5). 5.0 mg (6.43 µmol, 79%) of ester **13** were obtained as an off-white solid, that still contained triphenylphosphine oxide as an impurity. R_f (**13**) = 0.30 (silica gel, dichloromethane/MeOH 95:5). LCMS: Luna, MeCN/H_2O + 0.1% HCOOH 10:90, gradient 90:10, 0.8 mL/min, t_{R1} = 9.47 min ($[M + H]^+$ = 545), t_{R2} = 9.71 min ($[M + H]^+$ = 279 (Ph_3PO)).

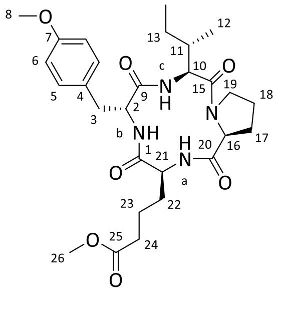

^{1}H NMR (400 MHz, $CDCl_3$): δ = 0.72 (t, $^3J_{14,13}$ = 7.2 Hz, 3 H, 14-H), 0.81 (d, $^3J_{12,11}$ = 6.4 Hz, 3 H, 12-H), 0.87 (m, 1 H, 13-H_a), 1.06 (m, 1 H, 13-H_b), 1.57 – 1.78 (m, 3 H, 22-H_a, 23-H), 1.83 – 1.94 (m, 3 H, 11-H, 18-H_a, 22-H_b), 1.98 (m, 1 H, 18-H_b), 2.21 (m, 2 H, 17-H), 2.33 (m, 2 H, 24-H), 2.83 (dd, $^2J_{3a,3b}$ = 13.5 Hz, $^3J_{3a,2}$ = 5.6 Hz, 1 H, 3-H_a), 3.19 (dd, $^2J_{3b,3a}$ = 13.3 Hz, $^3J_{3b,2}$ = 10.0 Hz, 1 H, 3-H_b), 3.67 (m, 1 H, 19-H_{eq}), 3.65 (s, 3 H, 26-H), 3.70 (s, 3 H, 8-H), 3.78 (dd, $^2J_{19ax,19eq}$ ≈ $^3J_{19ax,18ax}$ = 10.2 Hz, 1 H, 19-H_{ax}), 4.52 (dd, $^3J_{10,11}$ ≈ $^3J_{10,NH}$ = 9.7 Hz, 1 H, 10-H), 4.58 (m, 1 H, 21-H), 4.63 (m, 1 H, 2-H), 4.95 (d, $^3J_{16,17a/b}$ = 8.4 Hz, 1 H, 16-H), 6.21 (br s, 1 H, NH_a), 6.54 (br s, 1 H, NH_c), 6.66 (d, $^3J_{6,5}$ = 8.6 Hz, 2 H, 6-H), 6.86 (m, 1 H, NH_b), 7.01 (d, $^3J_{5,6}$ = 8.3 Hz, 2 H, 5-H) ppm. ^{13}C NMR (100 MHz, $CDCl_3$): δ = 11.3 (q, C-14), 15.9 (q, C-12), 21.0 (t, C-23), 21.9 (t, C-18), 24.1 (t, C-13), 27.6 (t, C-22), 31.6 (t, C-17), 33.3 (t, C-24), 34.5 (t, C-3), 36.3 (d, C-11), 47.1 (t, C-19), 51.6 (q, C-26), 53.7 (d, C-21), 55.1 (q, C-8), 55.7 (d, C-10), 57.1 (d, C-2), 61.0 (d, C-16), 113.7 (d, C-6), 128.4 (s, C-4), 130.4 (d, C-5), 158.3 (s, C-7), 170.1 (s, C-9), 170.5 (s, C-15), 173.3 (s, C-25), 173.5 (s, C-1), 174.8 (s, C-20). HRMS (CI) calculated for $C_{28}H_{41}N_4O_7$ $[M]^{++}$: 545.2970, found: 545.2952.

_S_-((4-(2-((3_S_,6_R_,9_S_,14a_S_)-9-((_S_)-_sec_-Butyl)-6-(4-methoxybenzyl)-1,4,7,10-tetraoxotetra-deca-hydropyrrolo[1,2-_a_][1,4,7,10]tetraazacyclododecin-3-yl)ethyl)tetrahydrofuran-3-yl)methyl) ethanethioate (14a)

To a suspension of 10.2 mg (18.4 µmol) **11** in 0.5 mL THF were added 2.63 µL (2.80 mg, 36.8 µmol) thioacetic acid and 6.0 µL (6.0 µmol) BEt_3 (1 M in THF) at room temperature. Then, 0.1 mL air were added and the reaction mixture was stirred at room temperature overnight before further 2.0 µL (2.13 mg, 28.0 µ mol) thioacetic acid and 6.0 µL (6.0 µmol) BEt_3 (1 M in THF) were added. Stirring was continued for 3 h and the reaction mixture was concentrated *in vacuo*. Purification by column chromatography (silica gel, dichloromethane/MeOH 100:0, gradient 95:5) afforded 9.5 mg (15.1 µmol, 82%) of compound **14** as an off-white solid, mp.: 238 °C (decomposition). R_f (**14a**) = 0.19 (silica gel, dichloromethane/MeOH 95:5). LCMS: Luna, MeCN/H_2O + 0.1% HCOOH 10:90, gradient 90:10, 0.8 mL/min, t_R = 9.88 min ($[M + H]^+$ = 631).

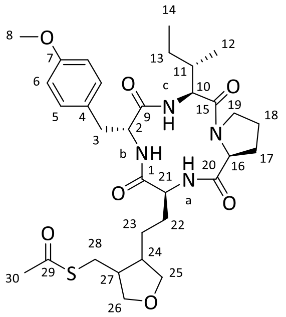

Major diastereomer: ^{1}H NMR (400 MHz, $CDCl_3$): δ = 0.72 (m, 3 H, 14-H), 0.80 (d, $^3J_{12,13}$ = 6.2 Hz, 3 H, 12-H), 0.82 (m, 1 H, 13-H_a), 1.04 (m, 1 H, 13-H_b), 1.27 (m, 2 H, 23-H), 1.71 (m, 1 H, 22-H_a), 1.80 – 1.94 (m, 4 H, 11-H, 18-H_a, 22-H_b, 24-H), 2.01 (m, 2 H, 18-H_b, 27-H), 2.22 (m, 2 H, 17-H), 2.33 (s, 3 H, 30-H), 2.78 – 2.87 (m, 2 H, 3-H_a, 28-H_a), 3.03 (m, 1 H, 28-H_b), 3.21 (m, 1 H, 3-H_b), 3.45 (m, 1 H, 26-H_a), 3.57 – 3.79 (m, 4 H, 19-H, 25-H), 3.71 (s, 3 H, 8-H), 3.88 (dd, $^2J_{26b,26a}$ ≈ $^3J_{26b,24/27}$ = 9.3 Hz, 1 H, 26-H_b), 4.49 – 4.68 (m, 3 H, 2-H, 10-H, 21-H), 4.93 (m, 1 H, 16-H), 6.26 (m, 1 H, NH_c), 6.46 (br s, 1 H, NH_a), 6.66 (d, $^3J_{6,5}$ = 8.6 Hz, 2 H, 6-H), 6.86 (m, 1 H, NH_b), 7.02 (m, 2 H, 5-H) ppm. ^{13}C NMR (100 MHz, $CDCl_3$): δ = 11.3 (q, C-14), 15.9 (q, C-12), 21.9 (t, C-18), 24.1 (t, C-13), 27.7 (t, C-22), 29.3 (t, C-23), 30.6 (q, C-30), 31.6 (t, C-17), 31.7 (t, C-28), 34.5 (t, C-3), 36.3 (d, C-24), 44.5 (d, C-27), 47.1 (t, C-19), 54.0 (d, C-21), 55.1 (q, C-8), 55.7 (d, C-10), 57.2 (d, C-2), 61.1 (d, C-16), 72.0, 72.6 (2 t, C-25/C-26), 113.7

(d, C-6), 128.6 (s, C-4), 130.4 (d, C-5), 158.3 (s, C-7), 170.1 (s, C-9), 170.6 (s, C-15), 195.5 (s, C-29) ppm. The signals of C-1, C-11 and C-20 weren't observed in the ^{13}C NMR spectrum. Minor diastereomer (selected signals): ^{1}H NMR (400 MHz, $CDCl_3$): δ = 2.34 (s, 3 H, 30-H), 2.64 (m, 1 H, 28-H_a), 3.36 (m, 1 H, 25-H_a), 3.69 (s, 3 H, 8-H), 3.70 (s, 3 H, 8-H), 3.82 (m, 1 H, 26-H_a), 3.97 (m, 1 H, 25-H_b), 6.65 (d, $^{3}J_{6,5}$ = 8.6 Hz, 2 H, 6-H), 6.67 (d, $^{3}J_{6,5}$ = 8.2 Hz, 2 H, 6-H), 6.68 (d, $^{3}J_{6,5}$ = 8.4 Hz, 2 H, 6-H). ^{13}C NMR (100 MHz, $CDCl_3$): δ = 11.3 (q, C-14), 11.3 (q, C-14), 11.4 (q, C-14), 15.9 (q, C-12), 15.9 (q, C-12), 21.9 (t, C-18), 24.1 (t, C-13), 27.6 (t, C-22), 29.3 (t, C-23), 44.6 (d, C-27), 47.2 (t, C-19), 55.1 (q, C-8), 130.5 (d, C-5), 195.4 (s, C-29) ppm. HRMS (CI) calculated for $C_{32}H_{47}N_4O_7S$ $[M + H]^+$: 631.3160, found: 631.3118.

***S*-((4-(2-((3*S*,6*R*,9*S*,14a*S*)-9-((*S*)-*sec*-Butyl)-6-(4-methoxybenzyl)-1,4,7,10-tetraoxotetra-deca-hydropyrrolo[1,2-*a*][1,4,7,10]tetraazacyclododecin-3-yl)ethyl)tetrahydrofuran-3-yl)methyl) benzothioate (14b)**

A suspension of 10.2 mg (18.4 µmol) **11** in 0.5 mL THF was treated with 4.34 µL (5.10 mg, 36.9 µmol) thiobenzoic acid and 6.0 µL (6.0 µmol) BEt_3 (1 M in THF) at room temperature. Then, 0.1 mL air were added and the reaction mixture was stirred at room temperature overnight before further 6.0 µL (6.0 µmol) BEt_3 (1 M in THF) were added and stirring was continued for 4 h. The reaction mixture was concentrated *in vacuo* and purified by column chromatography (silica gel, dichloromethane/MeOH 100:0, gradient 95:5) to yield 8.6 mg (12.4 µmol, 67%) of compound **14b** as an off-white solid, mp.: 208 °C (decomposition). R_f (**14b**) = 0.30 (silica gel, dichloromethane/MeOH 95:5). LCMS: Luna, MeCN/H_2O + 0.1% HCOOH 10:90, gradient 90:10, 0.8 mL/min, t_R = 10.8 min ($[M + H]^+$ = 693).

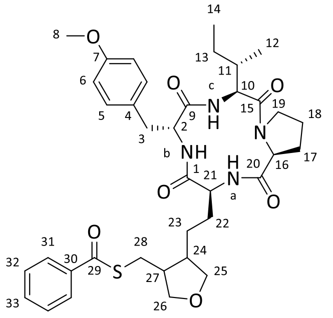

Major diastereomer: ^{1}H NMR (400 MHz, $CDCl_3$): δ = 0.71 (m, 3 H, 14-H), 0.80 (d, $^{3}J_{12,13}$ = 6.2 Hz, 3 H, 12-H), 0.83 (m, 1 H, 13-H_a), 1.04 (m, 1 H, 13-H_b), 1.31 (m, 1 H, 23-H_a), 1.58 (m, 1 H, 23-H_b), 1.76 (m, 1 H, 22-H_a), 1.82 – 2.04 (m, 5 H, 11-H, 18-H, 22-H_b, 28-H_a), 2.10 – 2.35 (m, 3 H, 17-H, 24-H), 2.47 (m, 1 H, 27-H), 2.85 (m, 1 H, 3-H_a), 3.14 – 3.27 (m, 2 H, 3-H_b, 28-H_b), 3.49 – 3.82 (m, 4 H, 19-H, 25-H_a, 26-H_a), 3.69 (s, 3 H, 8-H), 3.84 – 3.95 (m, 2 H, 25-H_b, 26-H_b), 4.49 – 4.71 (m, 3 H, 2-H, 10-H, 21-H), 5.00 (d, $^{3}J_{16,17a/b}$ = 6.4 Hz, 1 H, 16-H), 6.24 (m, 1 H, NH_a), 6.42 (m, 1 H, NH_c), 6.65 (d, $^{3}J_{6,5}$ = 7.0 Hz, 2 H, 6-H), 6.96 (m, 1 H, NH_b), 7.00 (d, $^{3}J_{5,6}$ = 7.7 Hz, 2 H, 5-H), 7.44 (dd, $^{3}J_{32,31} \approx {}^{3}J_{32,33}$ = 7.6 Hz, 2 H, 32-H), 7.57 (t, $^{3}J_{33,32}$ = 7.4 Hz, 1 H, 33-H), 7.93 (d, $^{3}J_{31,32}$ = 7.5 Hz, 2 H, 31-H) ppm. ^{13}C NMR (100 MHz, $CDCl_3$): δ = 11.3 (q, C-14), 15.8 (q, C-12), 21.9 (t, C-18), 23.7 (t, C-22), 24.1 (t, C-13), 27.4 (t, C-28), 29.7 (t, C-23), 31.7 (t, C-17), 34.6 (t, C-3), 36.3 (d, C-11), 41.7 (d, C-27), 44.7 (d, C-24), 47.0 (t, C-19), 53.9 (d, C-21), 55.0 (q, C-8), 55.8 (d, C-10), 57.3 (d, C-2), 61.0 (d, C-16), 70.6 (t, C-26), 72.3 (t, C-25), 113.7 (d, C-6), 127.2 (d, C-31), 128.6 (s, C-4), 128.7 (d, C-32), 130.5 (d, C-5), 133.5 (d, C-33), 136.8 (s, C-30), 158.3 (s, C-7), 170.2 (s, C-9), 170.6 (s, C-15), 173.6, 174.7 (2 s, C-1/C-20), 191.5 (s, C-29) ppm. Minor diastereomer (selected signals): ^{1}H NMR (400 MHz, $CDCl_3$): δ = 3.01 (m, 1 H, 28-H_b), 3.39 (m, 1 H, 25-H_a), 3.67 (s, 3 H, 8-H), 3.68 (s, 3 H, 8-H), 4.00 (dt, $^{2}J_{25b,25a}$ = 8.2 Hz, $^{3}J_{25b,24}$ = 7.5 Hz, 1 H, 25-H_b), 6.63 (d, $^{3}J_{6,5}$ = 6.0 Hz, 2 H, 6-H), 6.98 (d, $^{3}J_{5,6}$ =

6.1 Hz, 2 H, 5-H) ppm. ^{13}C NMR (100 MHz, $CDCl_3$): δ = 11.3 (q, C-14), 15.9 (q, C-12), 22.0 (t, C-18), 27.5 (t, C-28), 31.6 (t, C-17), 31.7 (t, C-17), 41.8 (d, C-27), 42.2 (d, C-27), 42.3 (d, C-27), 44.8 (d, C-24), 45.2 (d, C-24), 45.3 (d, C-24), 47.1 (t, C-19), 54.0 (d, C-21), 54.2 (d, C-21), 57.5 (d, C-2), 61.0 (d, C-16), 71.9 (d, C-25 o. C-26), 72.0 (d, C-25 o. C-26), 72.3 (t, C-25), 72.7 (2 d, C-25/C-26), 133.6 (d, C-33), 136.7 (s, C-30), 174.8, 174.9 (2 s, C-1/C-20), 191.4 (s, C-29) ppm. HRMS (CI) calculated for $C_{37}H_{49}N_4O_7S$ $[M]^+$: 693.3316, found: 693.3307.

References

1. Servatius, P.; Kazmaier, U. Org. Biomol. Chem. 2018, 16, 3464–3472.

Copies of NMR spectra

((4*S*,5*S*)-5-((Allyloxy)methyl)-2,2-dimethyl-1,3-dioxolan-4-yl)methanol (2)

^{1}H NMR (400 MHz, $CDCl_3$)

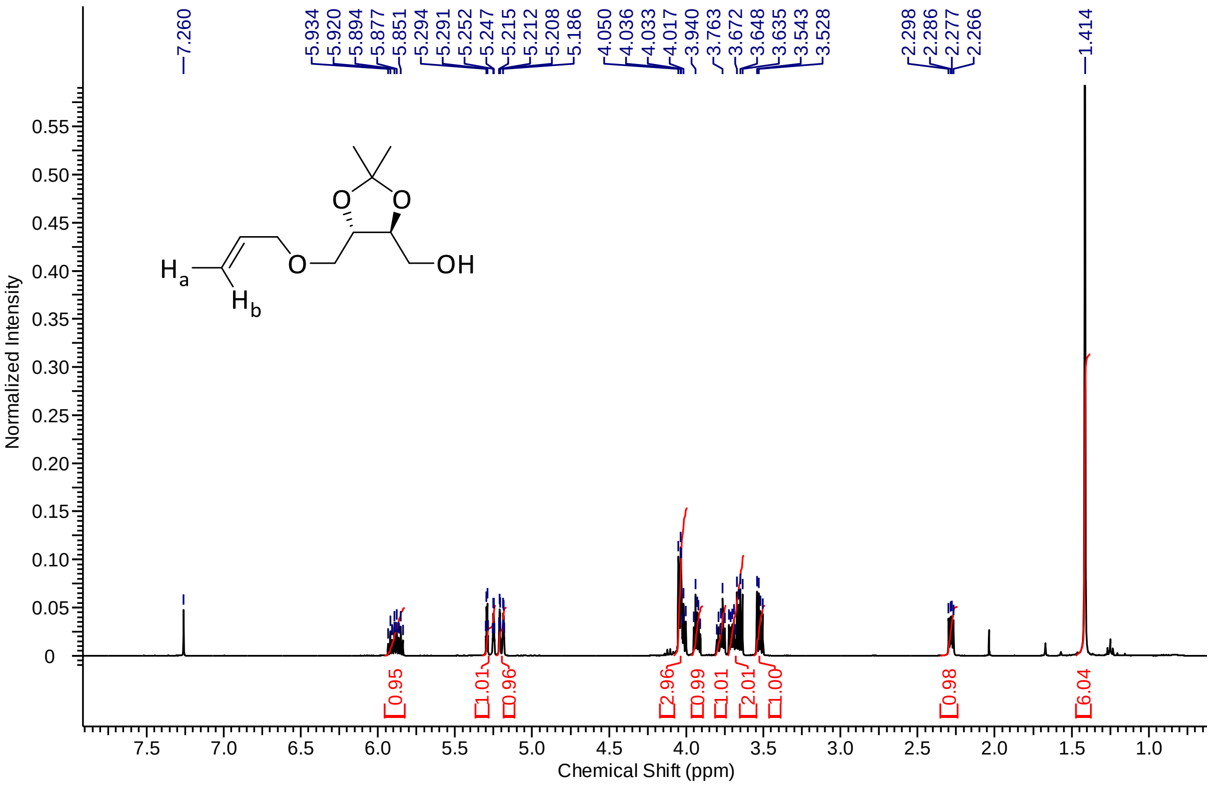

^{13}C NMR (100 MHz, $CDCl_3$)

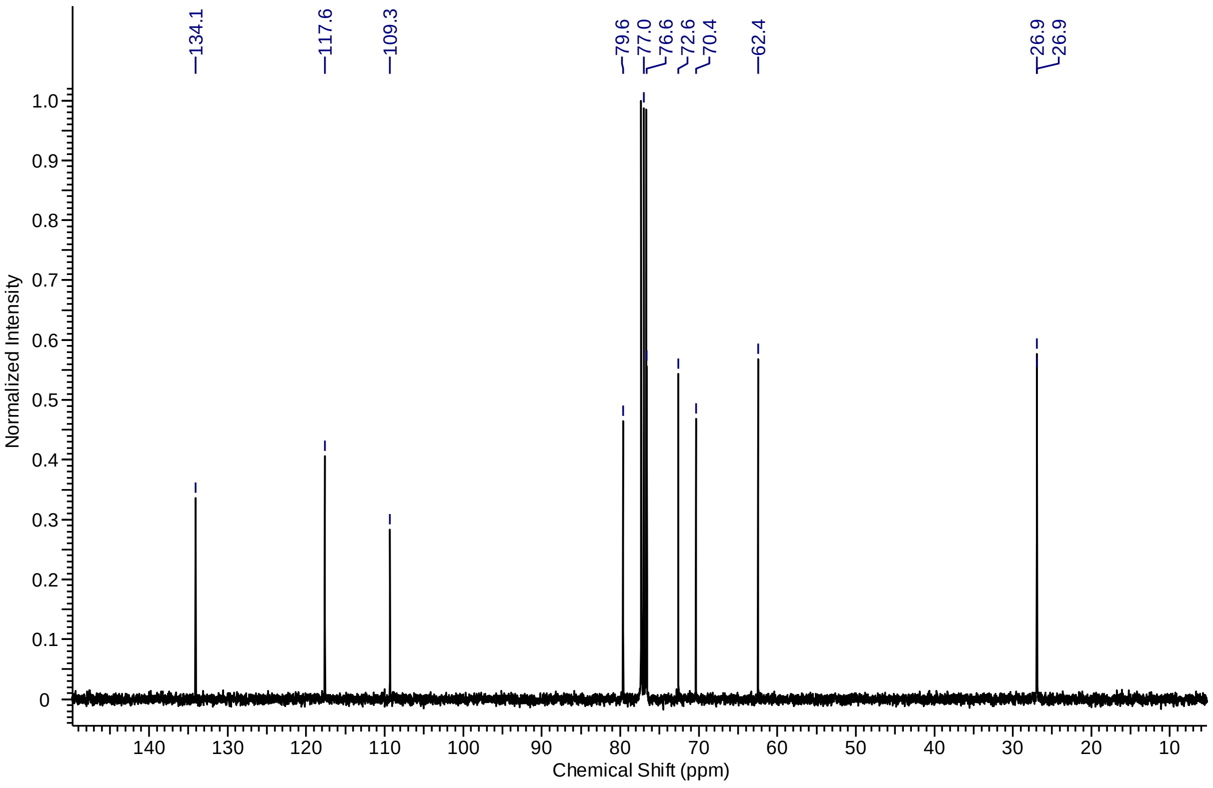

(4*S*,5*R*)-4-((Allyloxy)methyl)-5-(iodomethyl)-2,2-dimethyl-1,3-dioxolane (3)

^{1}H NMR (400 MHz, $CDCl_3$)

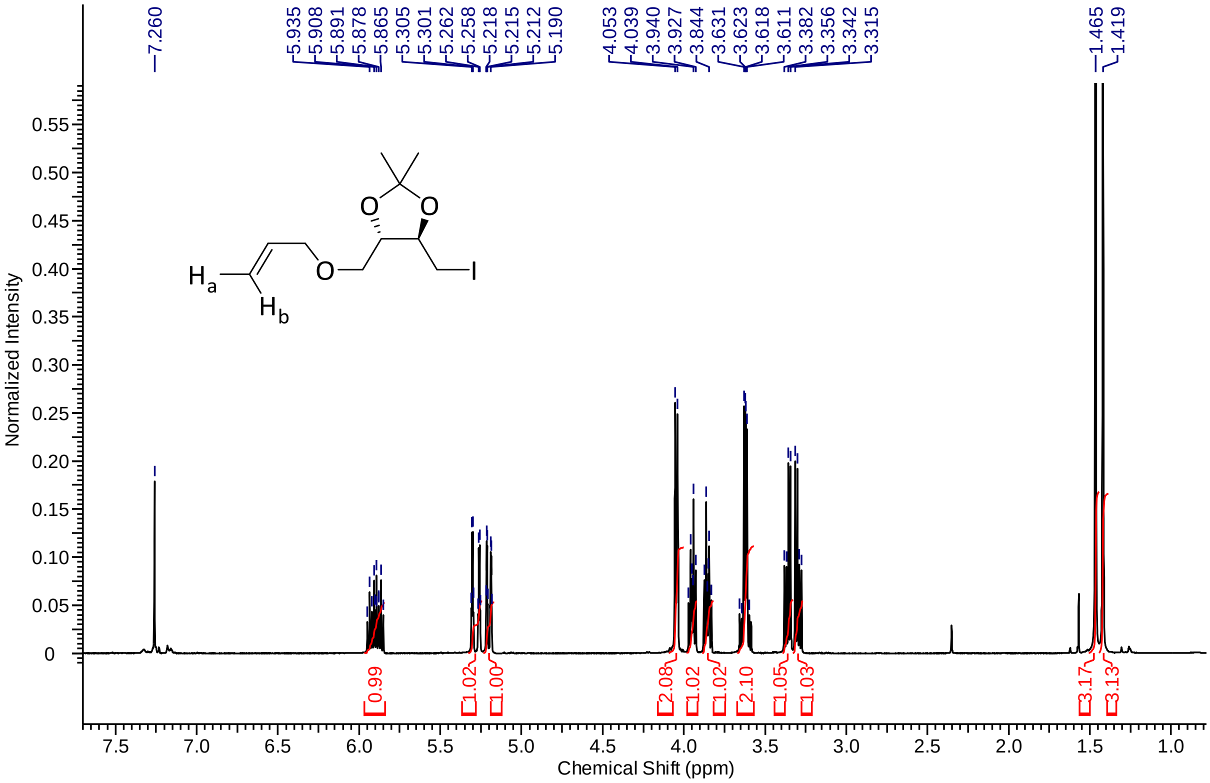

^{13}C NMR (100 MHz, $CDCl_3$)

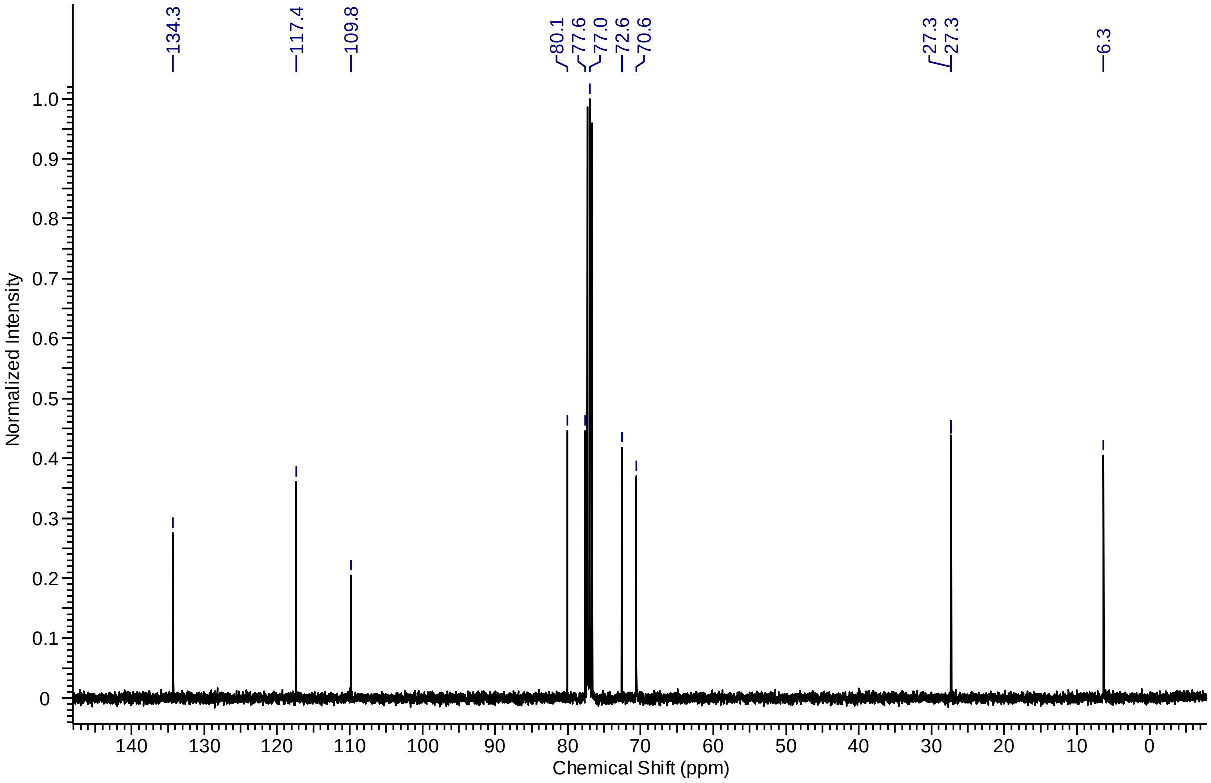

(*R*)-1-(Allyloxy)but-3-en-2-ol (4)

^{1}H NMR (400 MHz, $CDCl_3$)

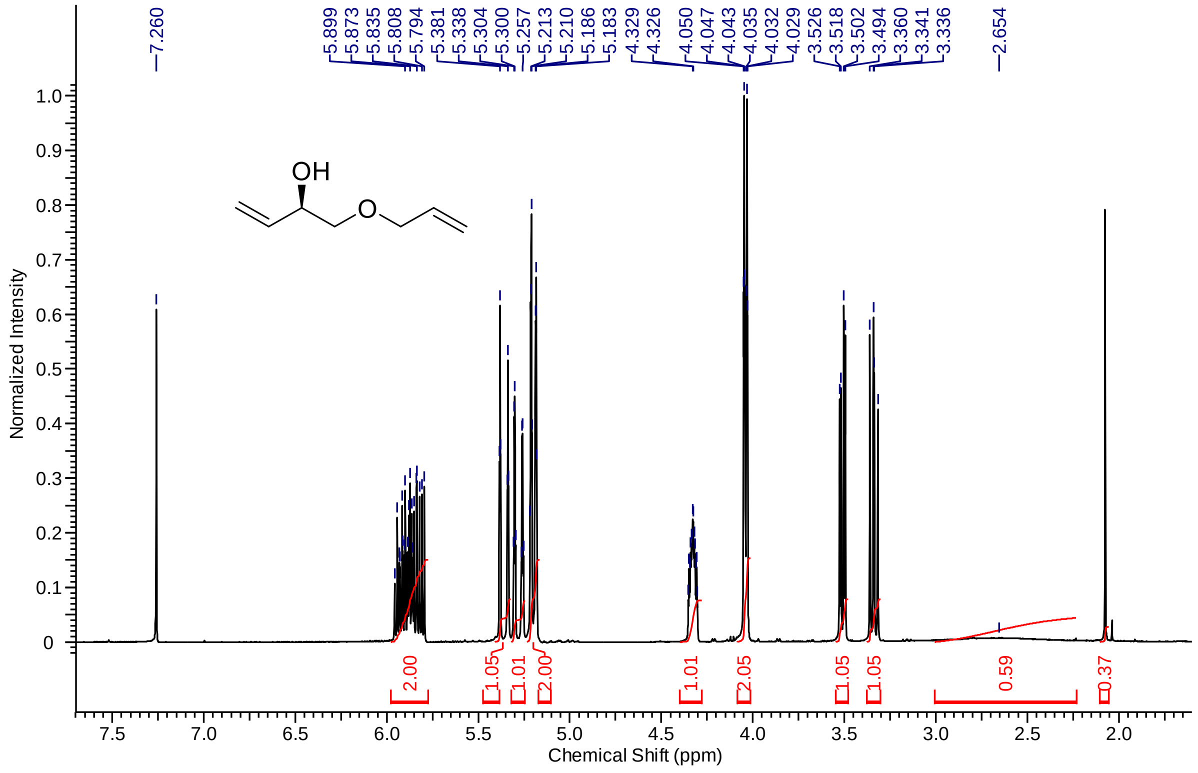

^{13}C NMR (100 MHz, $CDCl_3$)

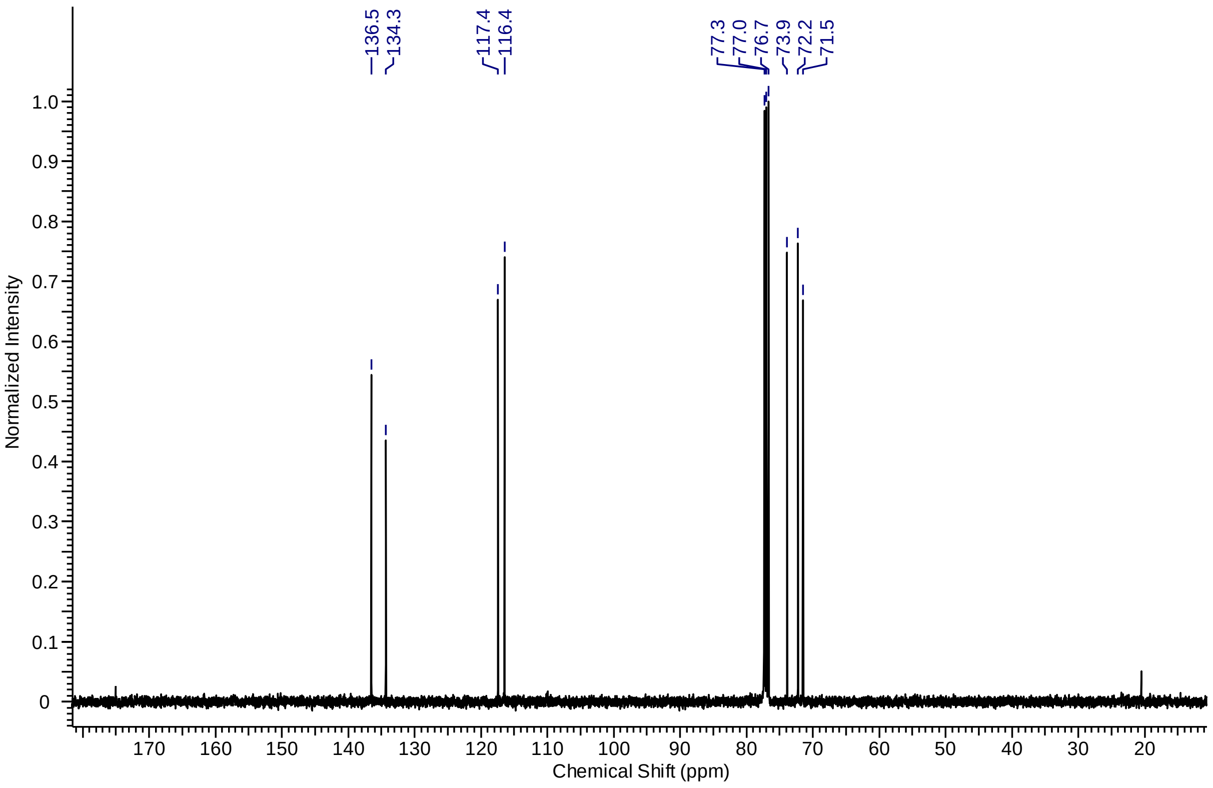

(*R*)-1-(Allyloxy)but-3-en-2-yl (*tert*-butoxycarbonyl)glycinate (5)

^{1}H NMR (400 MHz, CDCl$_3$)

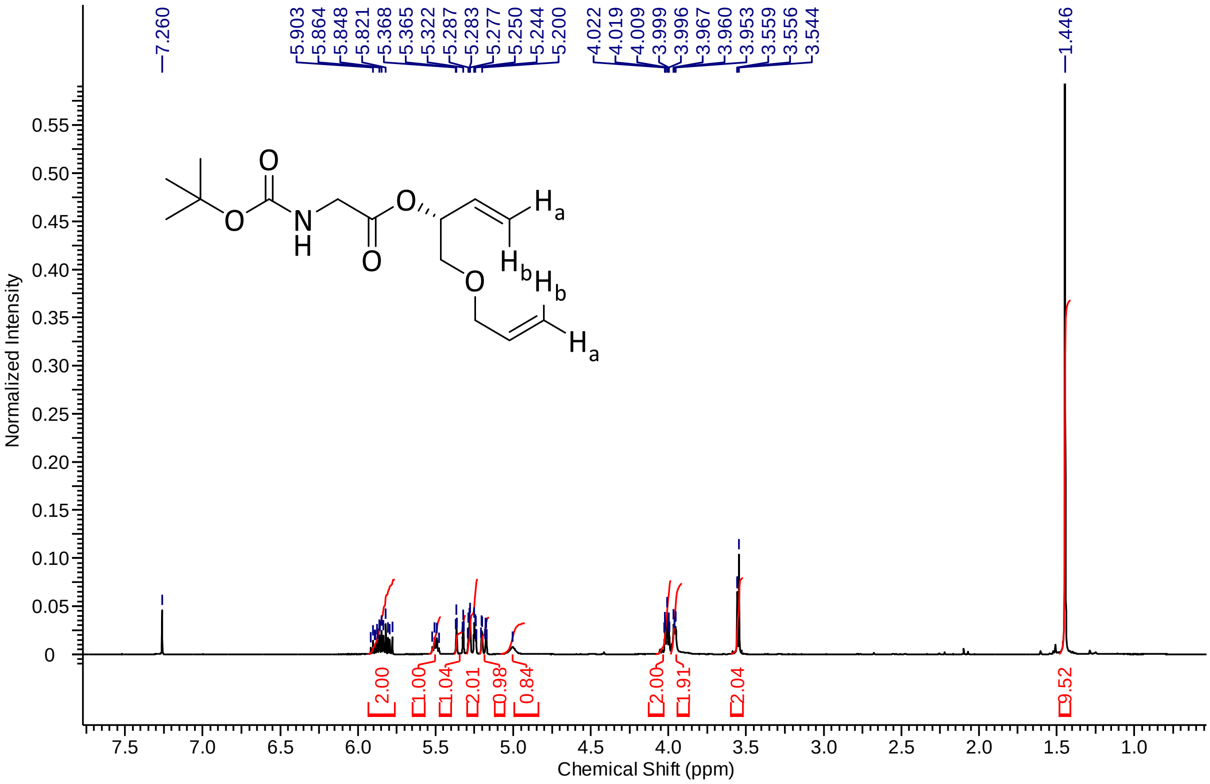

^{13}C NMR (100 MHz, CDCl$_3$)

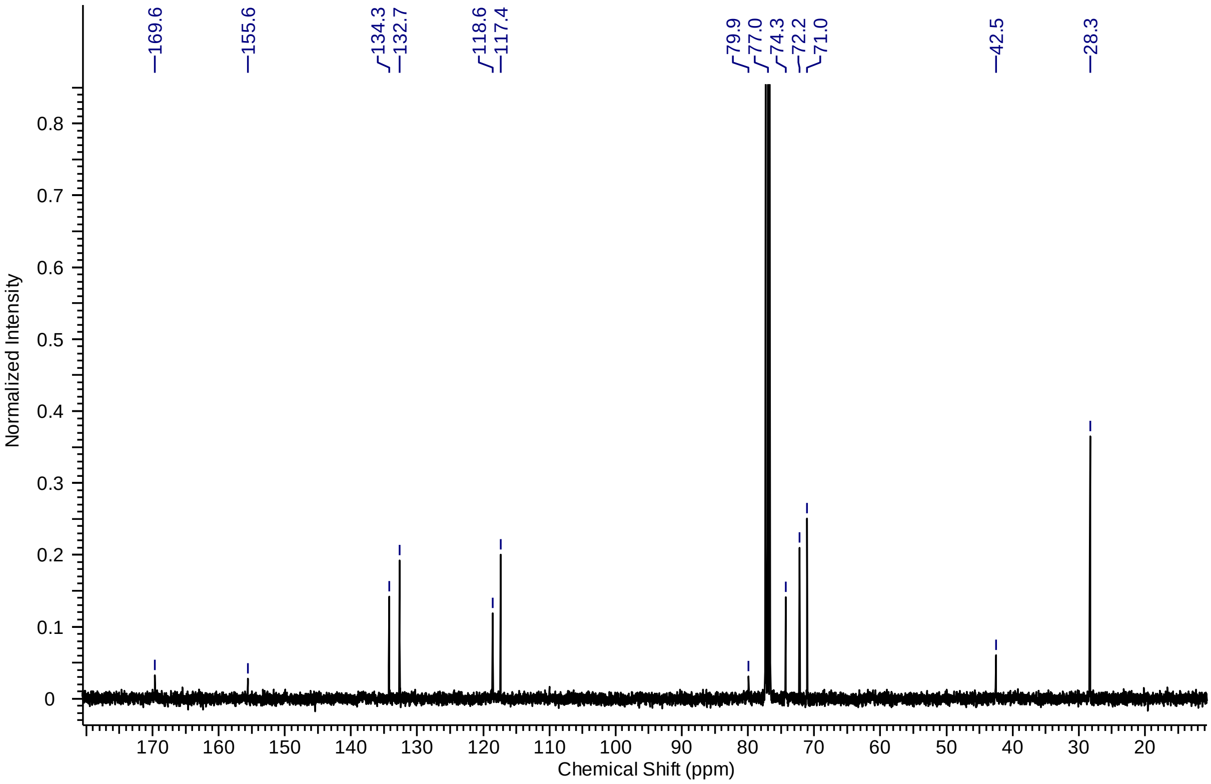

(*S,E*)-6-(Allyloxy)-2-((*tert*-butoxycarbonyl)amino)hex-4-enoic acid (6)

^{1}H NMR (400 MHz, $CDCl_3$)

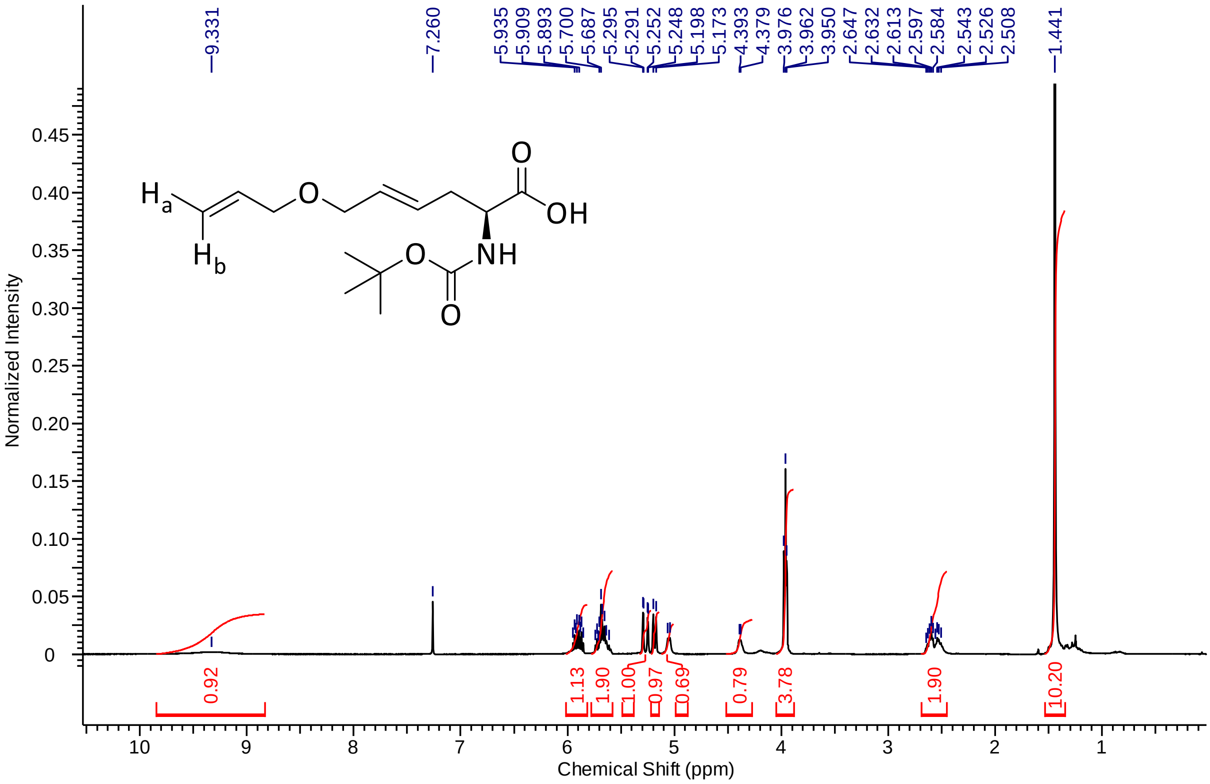

^{13}C NMR (100 MHz, $CDCl_3$)

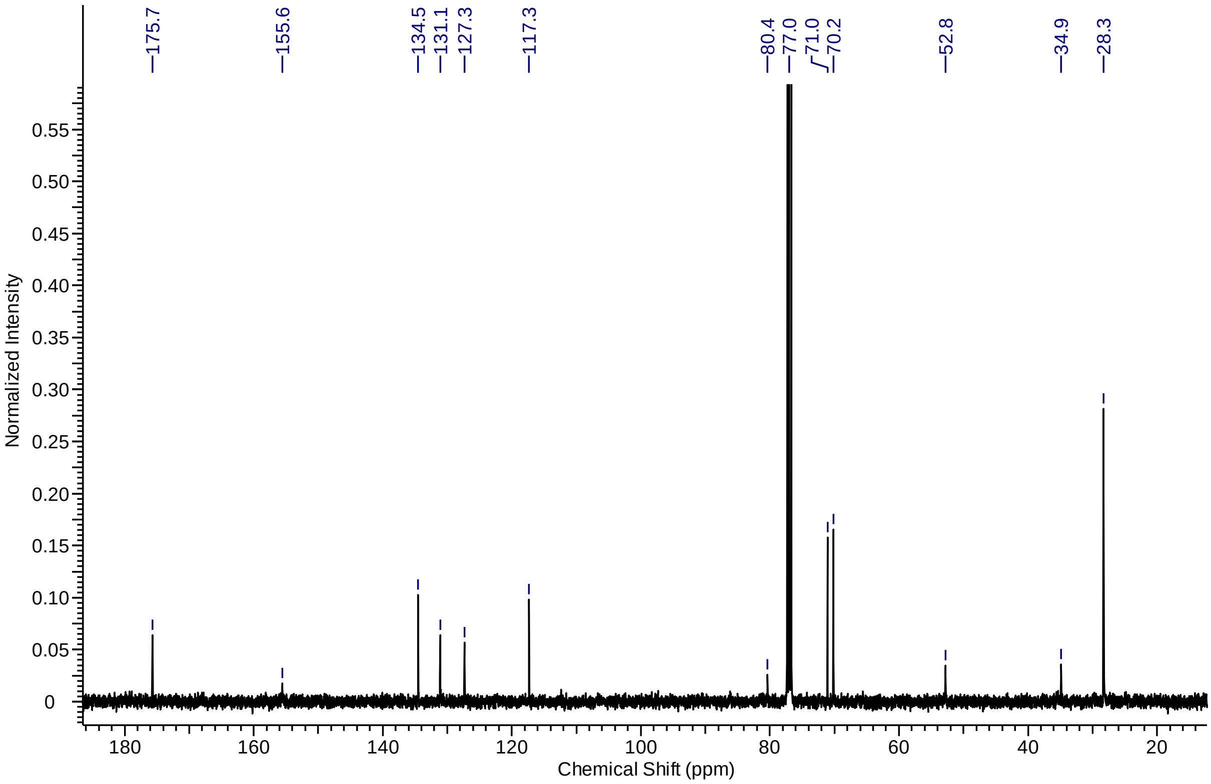

(*R*)-1-(Allyloxy)but-3-en-2-yl glycinate hydrochloride (7)

^{1}H NMR (400 MHz, $CDCl_3$)

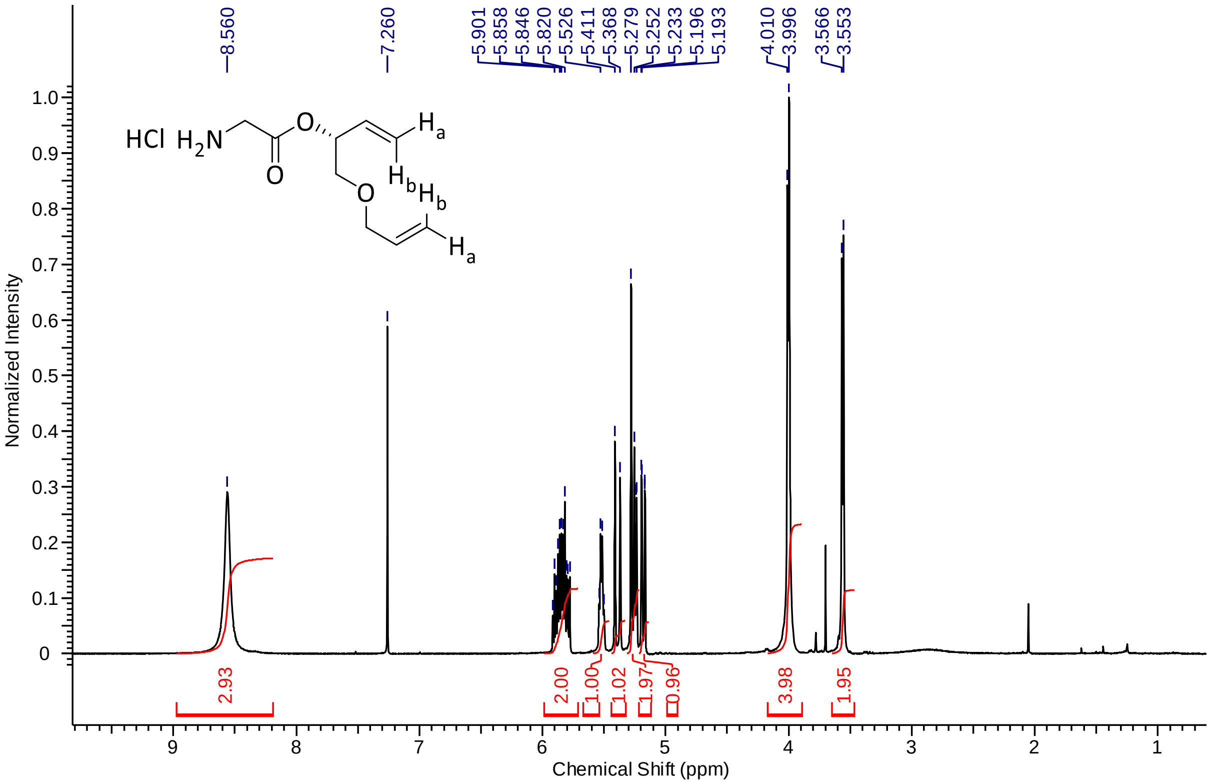

^{13}C NMR (100 MHz, $CDCl_3$)

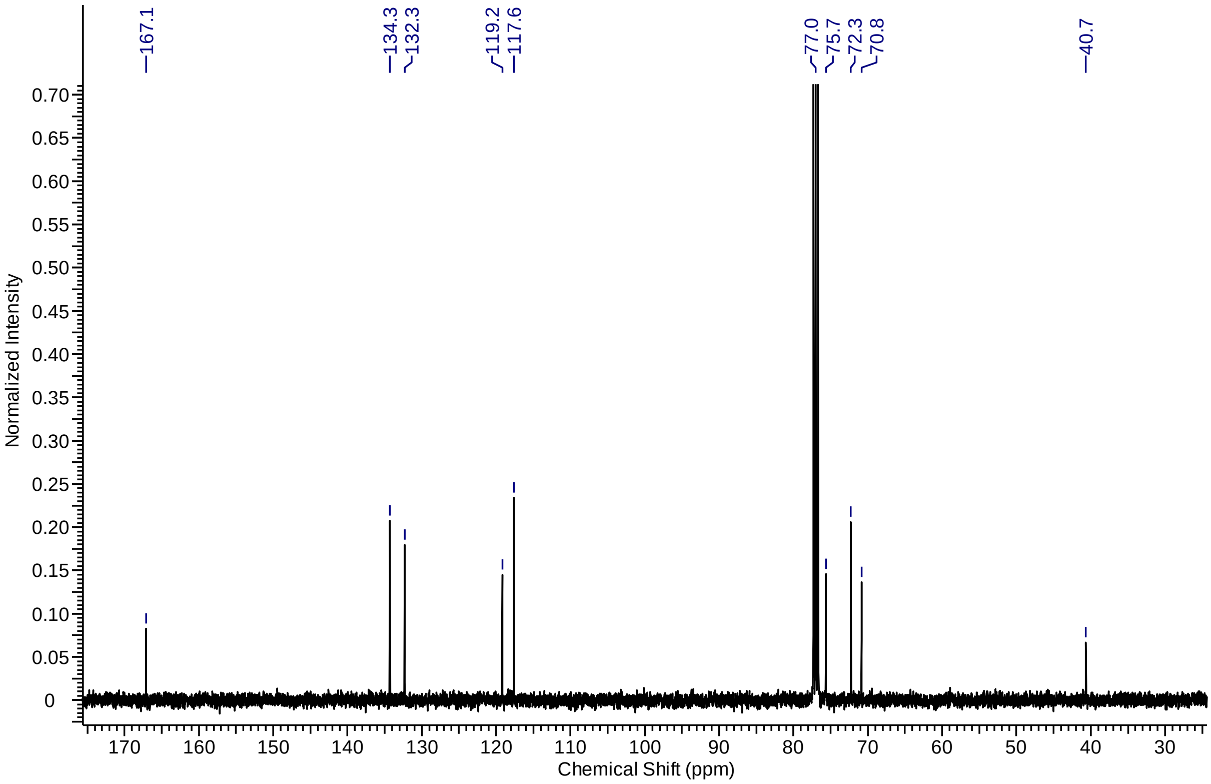

(*R*)-1-(Allyloxy)but-3-en-2-yl ((R)-2-(((benzyloxy)carbonyl)amino)-3-(4-methoxyphenyl)-propanoyl)-L-isoleucyl-L-prolylglycinate (8a)

^{1}H NMR (400 MHz, $CDCl_3$)

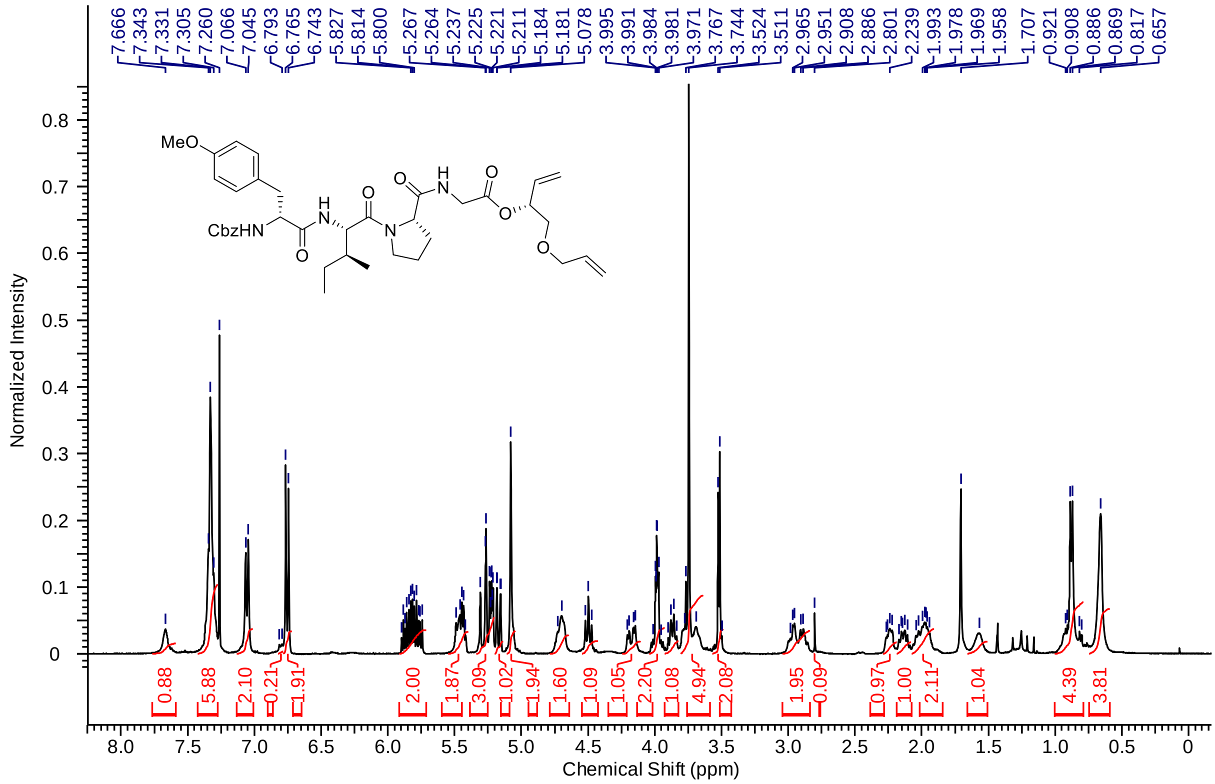

^{13}C NMR (100 MHz, $CDCl_3$)

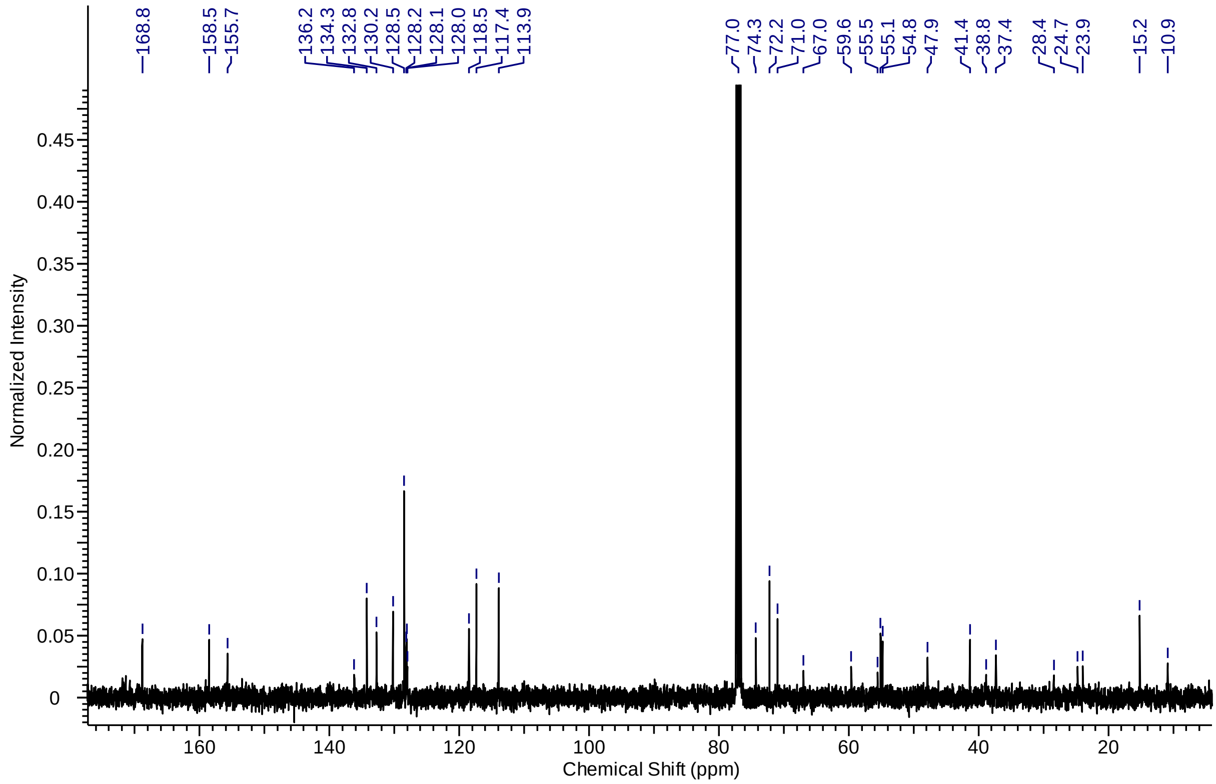

(*R*)-1-(Allyloxy)but-3-en-2-yl ((*R*)-2-((*tert*-butoxycarbonyl)amino)-3-(4-methoxyphenyl)-propanoyl)-ʟ-isoleucyl-ʟ-prolyl-glycinate (8b).

^{1}H NMR (400 MHz, $CDCl_3$)

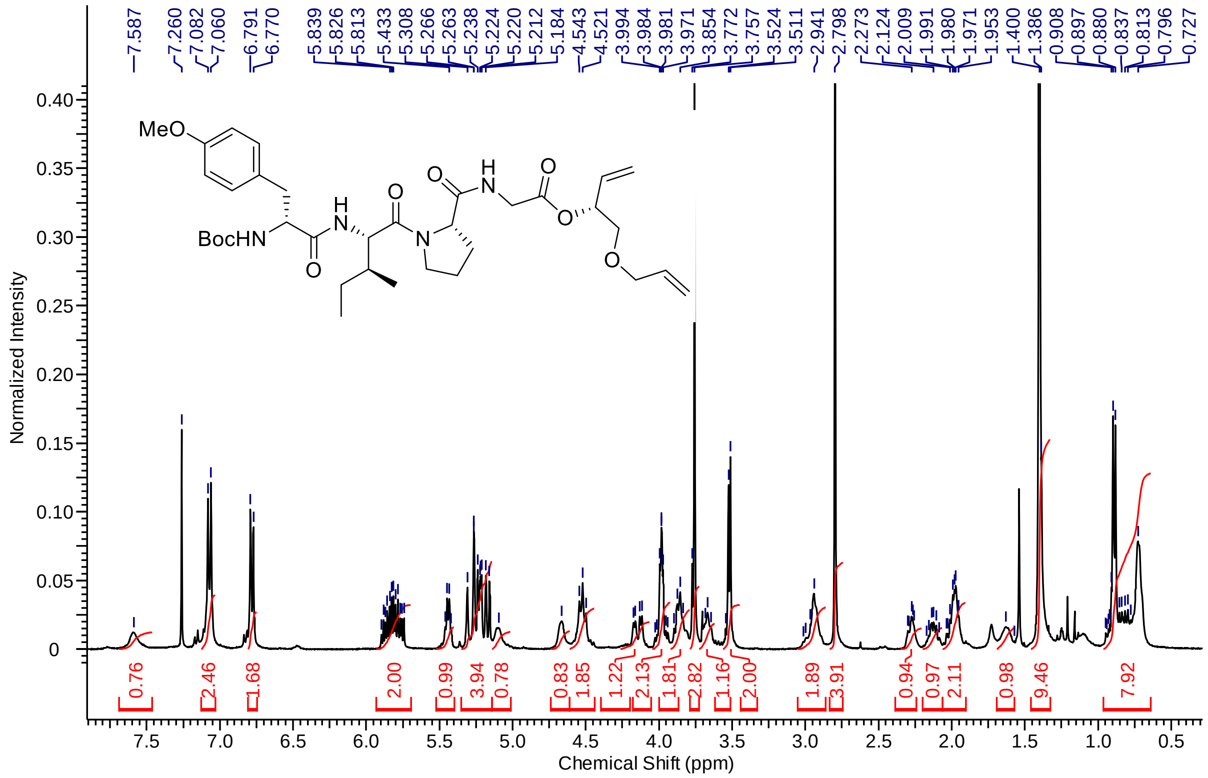

^{13}C NMR (100 MHz, $CDCl_3$)

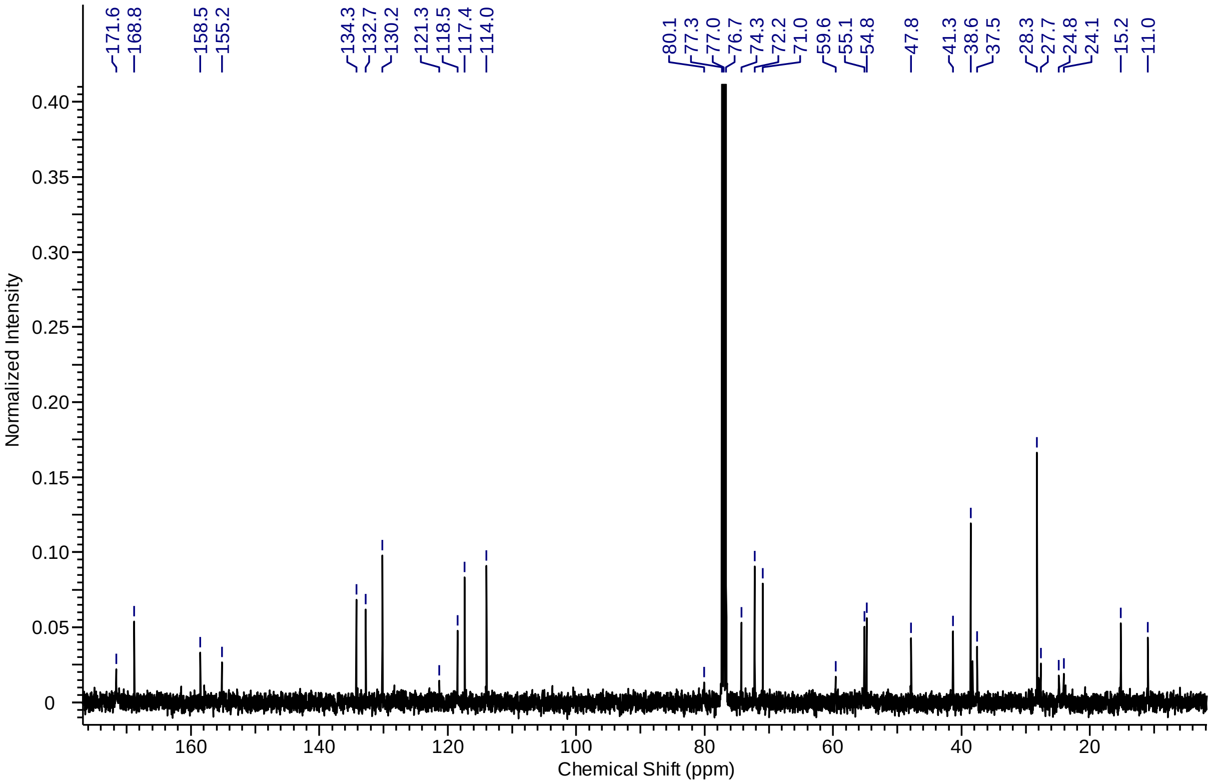

(*S,E*)-6-(Allyloxy)-2-((*S*)-1-(((*R*)-2-((*tert*-butoxycarbonyl)amino)-3-(4-methoxyphenyl)-pro-panoyl)-L-isoleucyl)pyrrolidine-2-carboxamido)hex-4-enoic acid (9b).

^{1}H NMR (400 MHz, $CDCl_3$)

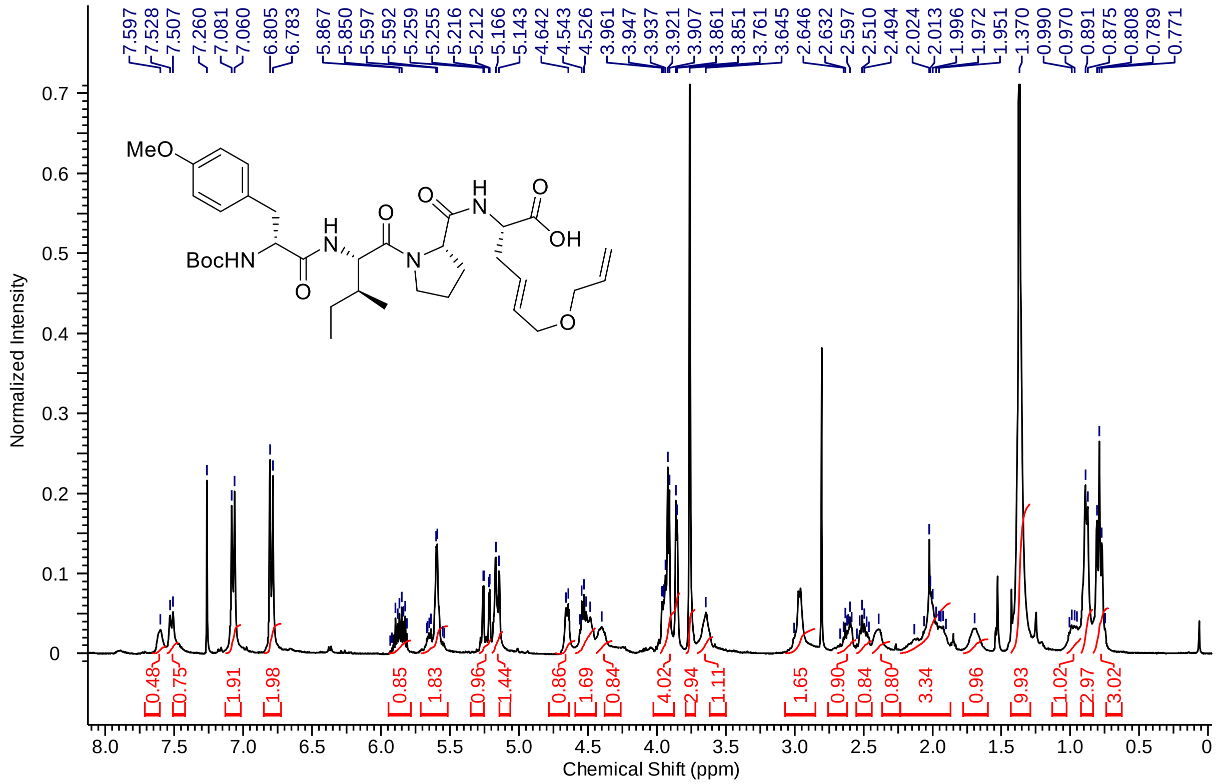

^{13}C NMR (100 MHz, $CDCl_3$)

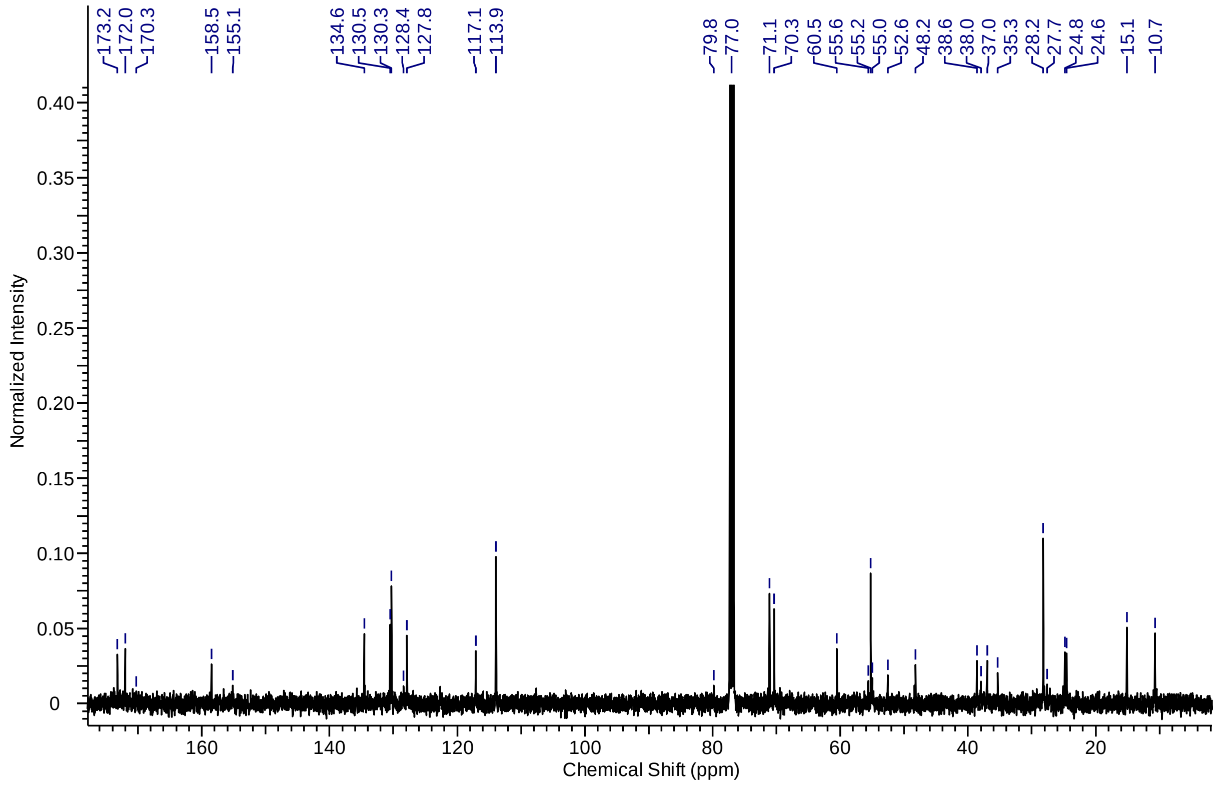

Pentafluorophenyl (*S,E*)-6-(allyloxy)-2-((*S*)-1-(((*R*)-2-((*tert*-butoxycarbonyl)amino)-3-(4-methoxyphenyl)propanoyl)-L-isoleucyl)pyrrolidine-2-carboxamido)hex-4-enoate (10)

^{1}H NMR (500 MHz, 373 K, DMSO-d_6)

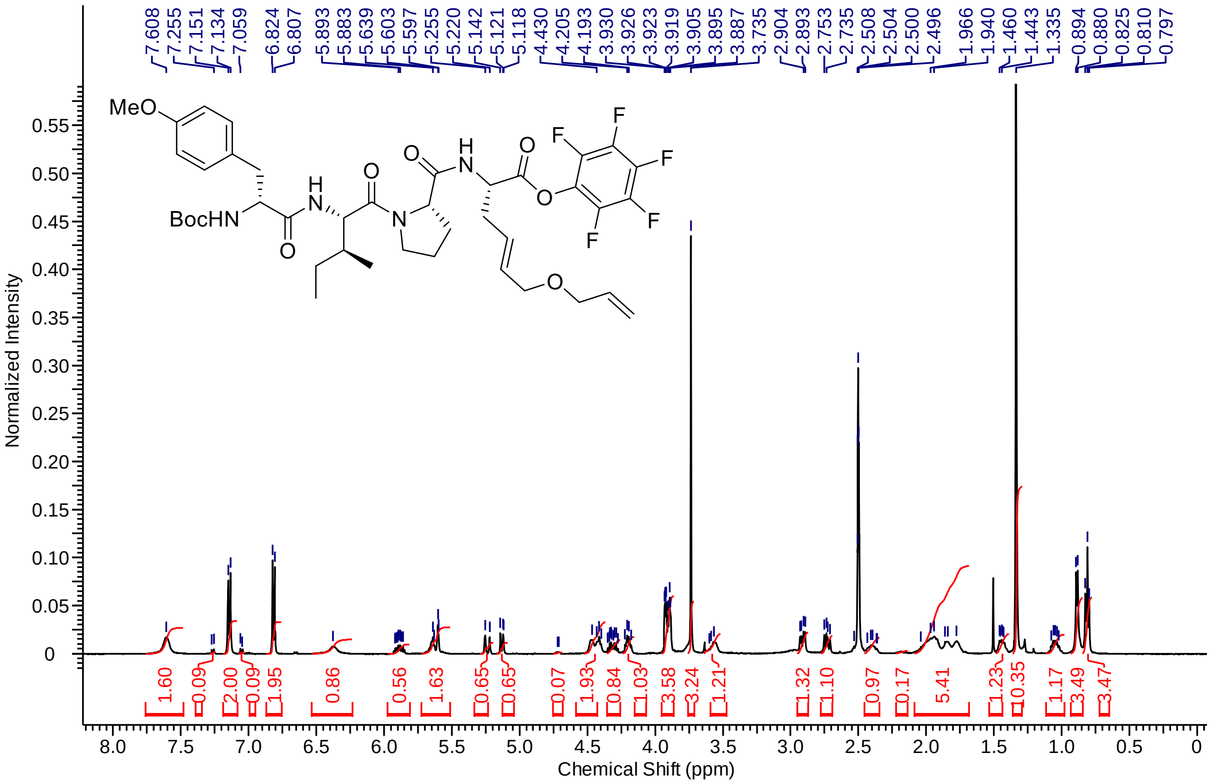

^{13}C NMR (100 MHz, $CDCl_3$)

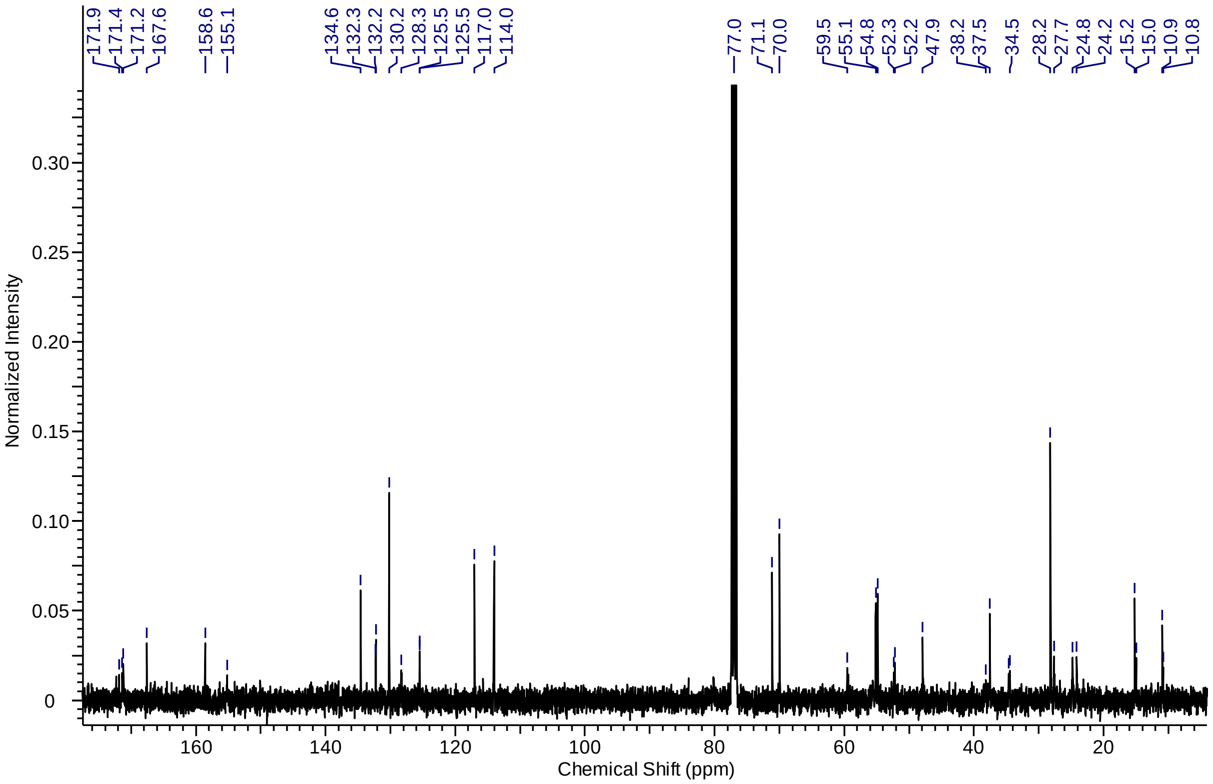

(3*S*,6*R*,9*S*,14a*S*)-3-((*E*)-4-(Allyloxy)but-2-en-1-yl)-9-((*S*)-*sec*-butyl)-6-(4-methoxybenzyl)-deca-hydropyrrolo[1,2-*a*][1,4,7,10]tetraazacyclodo-decine-1,4,7,10-tetraone (11)

^{1}H NMR (500 MHz, $CDCl_3$)

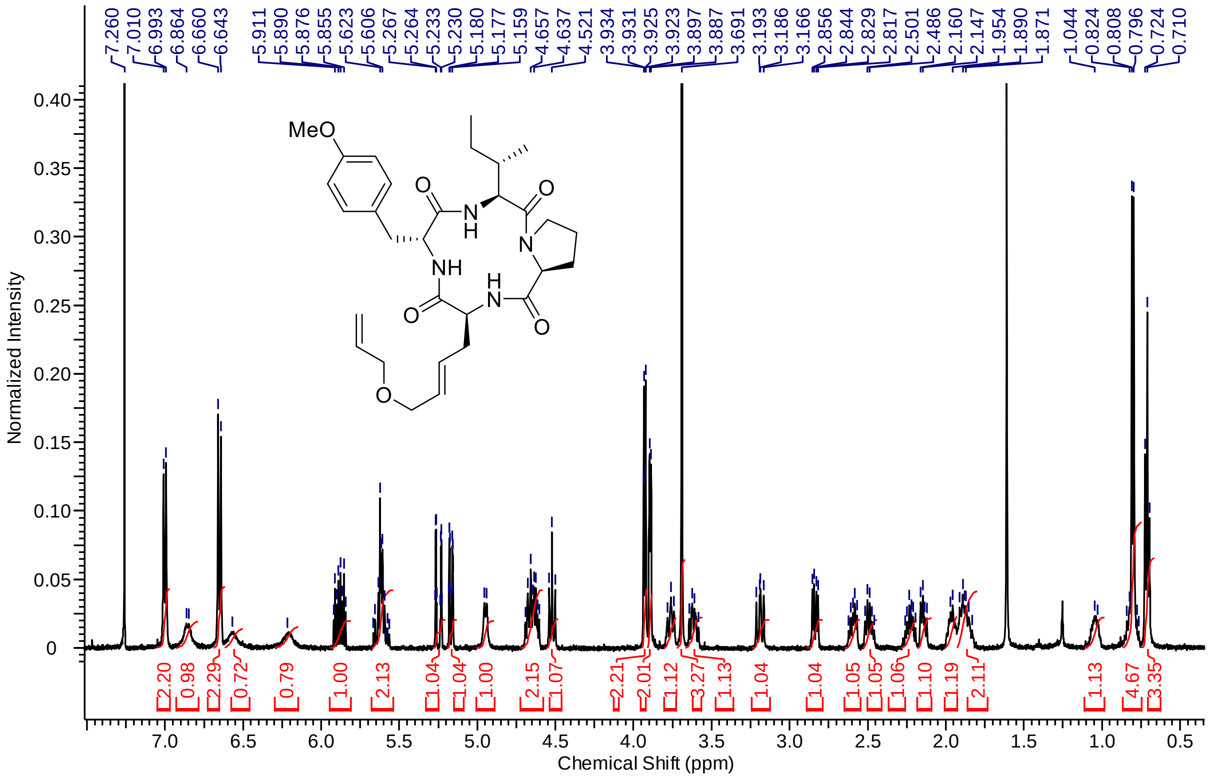

^{13}C NMR (125 MHz, $CDCl_3$)

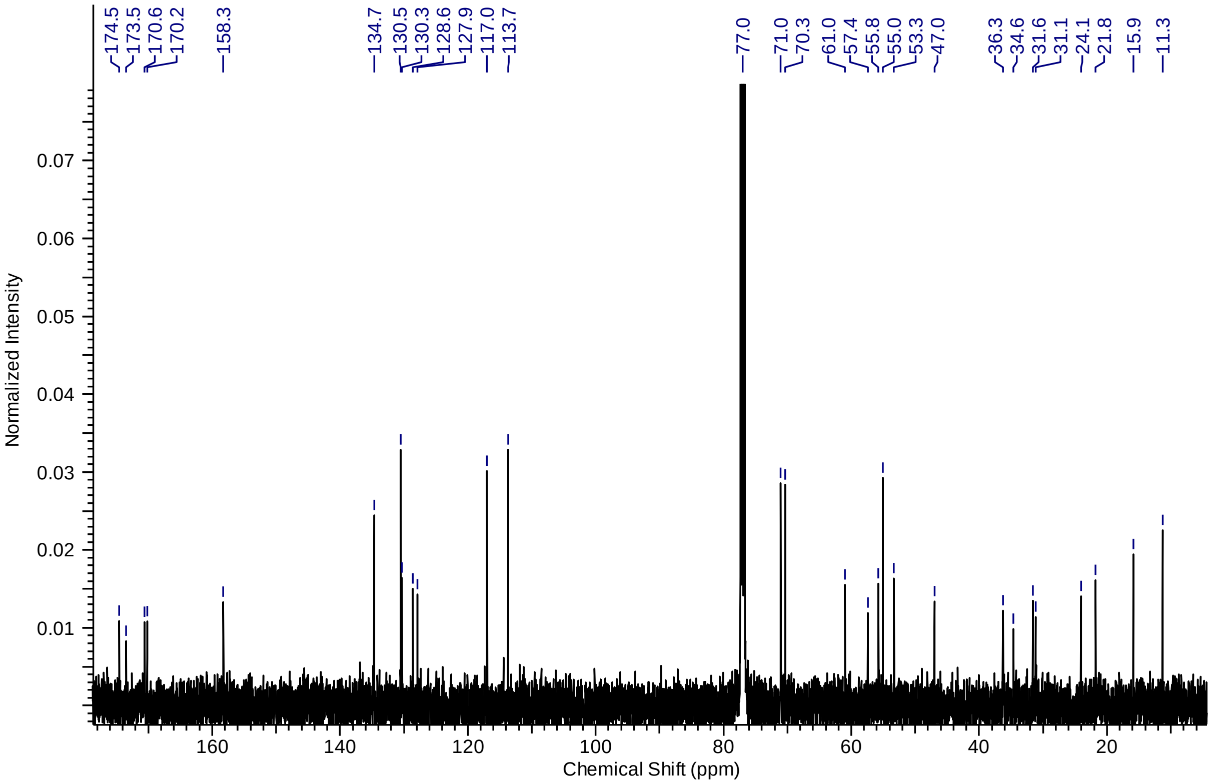

(3*S*,6*R*,9*S*,14a*S*)-3-allyl-9-((*S*)-*sec*-butyl)-6-(4-methoxybenzyl)decahydropyrrolo[1,2-*a*]-[1,4,7,10]tetraazacyclododecine-1,4,7,10-tetraone (12)

^{1}H NMR (500 MHz, 373 K, tetrachloroethane-d_2)

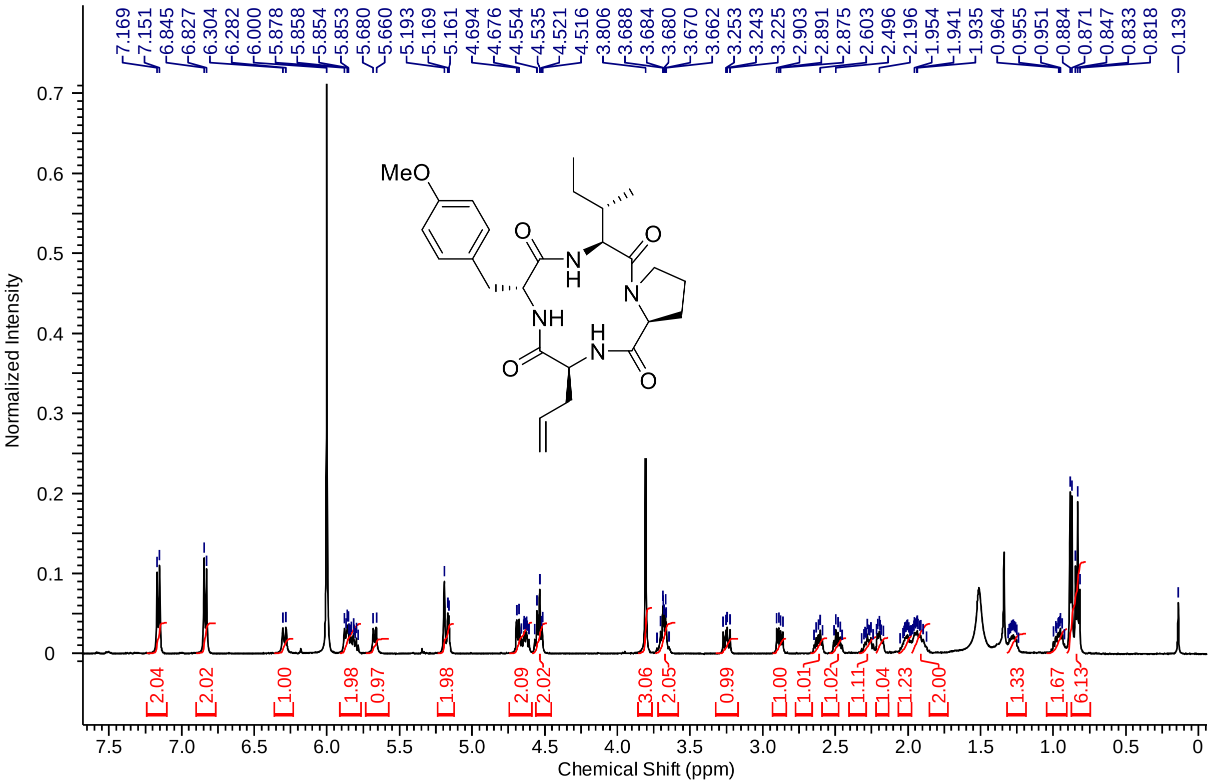

^{13}C NMR (125 MHz, 373 K, tetrachloroethane-d_2)

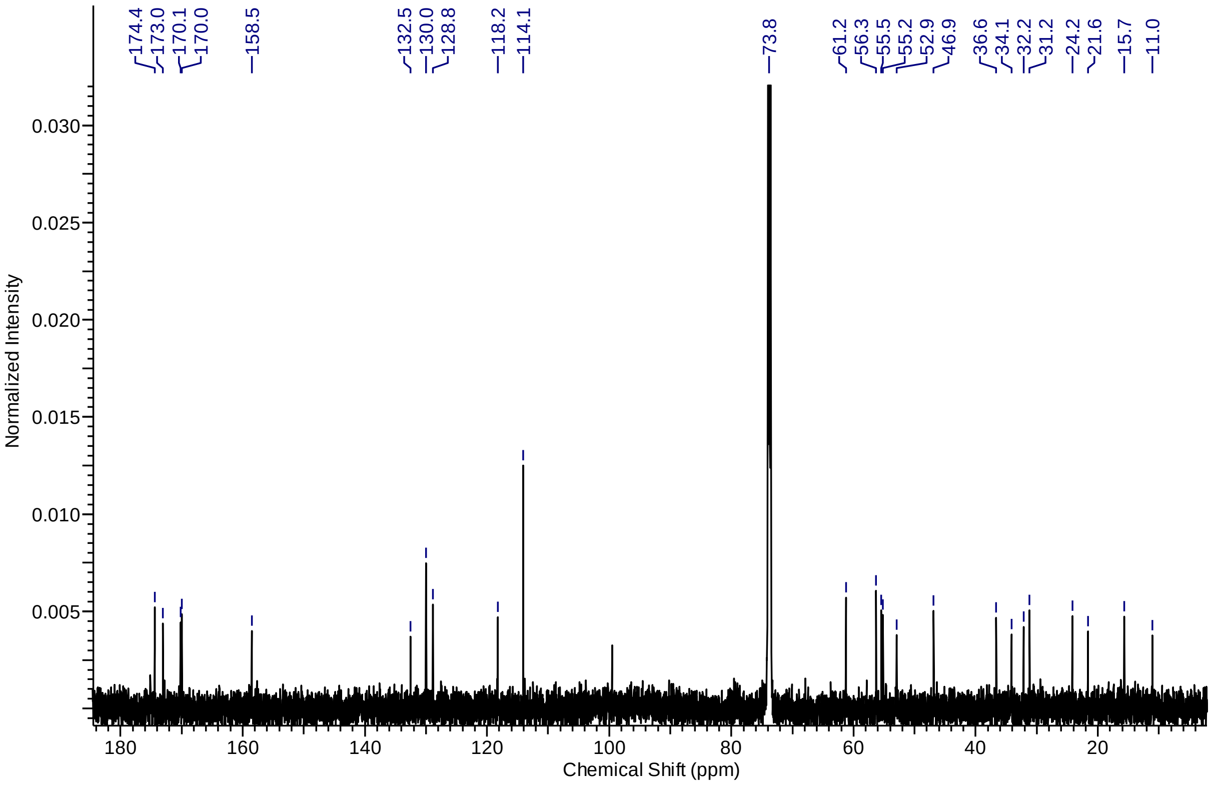

Methyl 4-((3*S*,6*R*,9*S*,14a*S*)-9-((*S*)-*sec*-butyl)-6-(4-methoxybenzyl)-1,4,7,10-tetraoxotetradecahydropyrrolo[1,2-*a*][1,4,7,10]tetraazacyclododecin-3-yl)butanoate (13)

^{1}H NMR (400 MHz, $CDCl_3$)

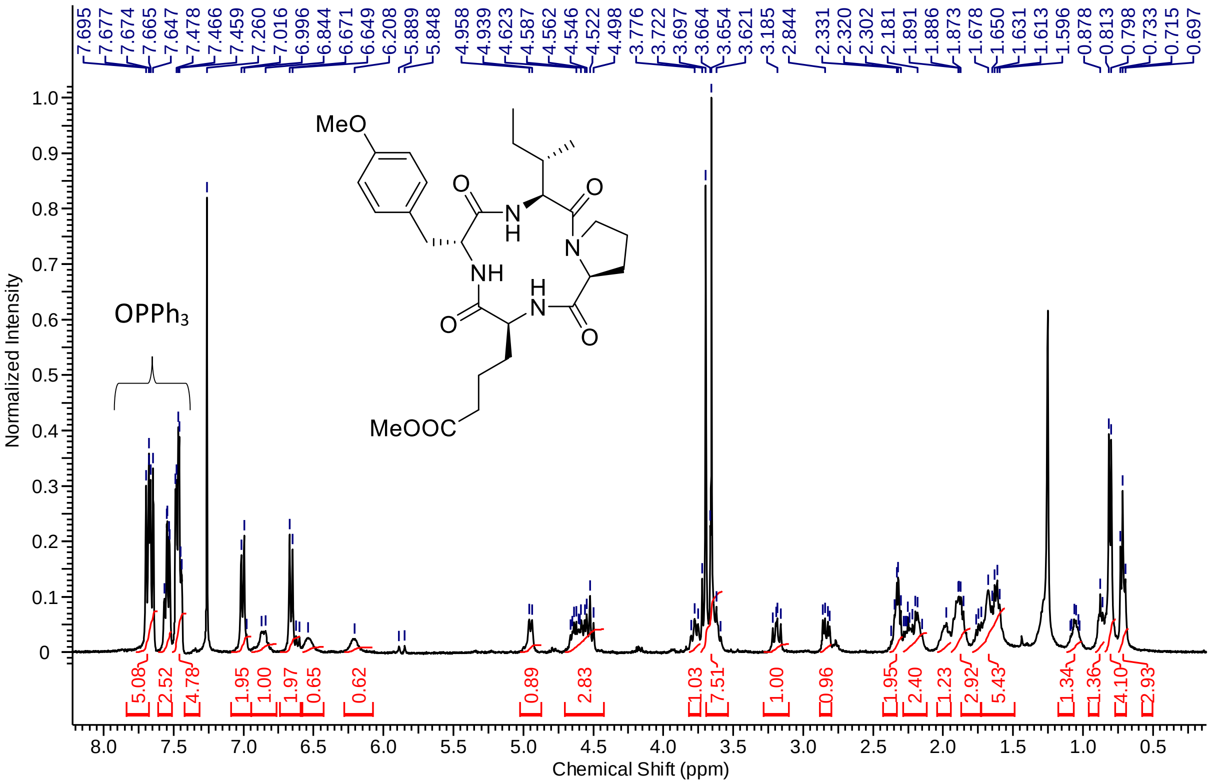

^{13}C NMR (100 MHz, $CDCl_3$)

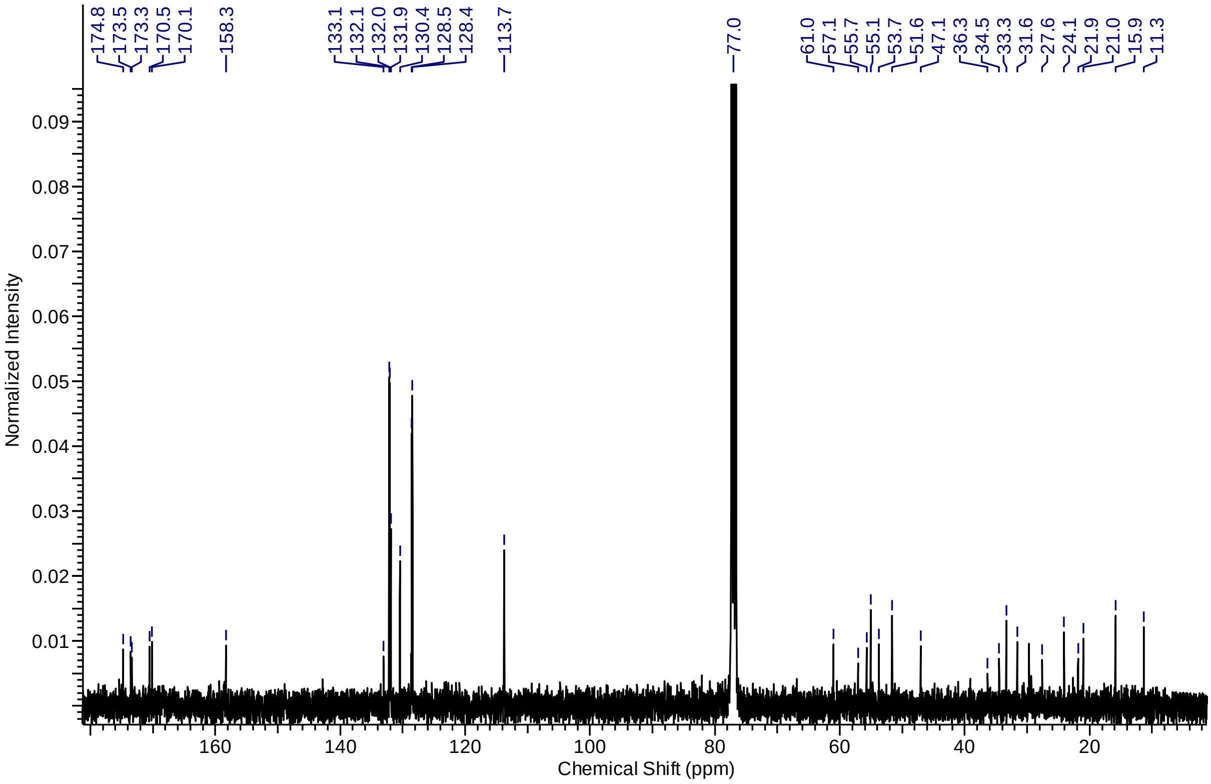

_S_-((4-(2-((3_S_,6_R_,9_S_,14a_S_)-9-((_S_)-_sec_-Butyl)-6-(4-methoxybenzyl)-1,4,7,10-tetraoxotetra-deca-hydropyrrolo[1,2-_a_][1,4,7,10]tetraazacyclodo-decin-3-yl)ethyl)tetrahydrofuran-3-yl)methyl) ethanethioate (14a)

^{1}H NMR (400 MHz, $CDCl_3$)

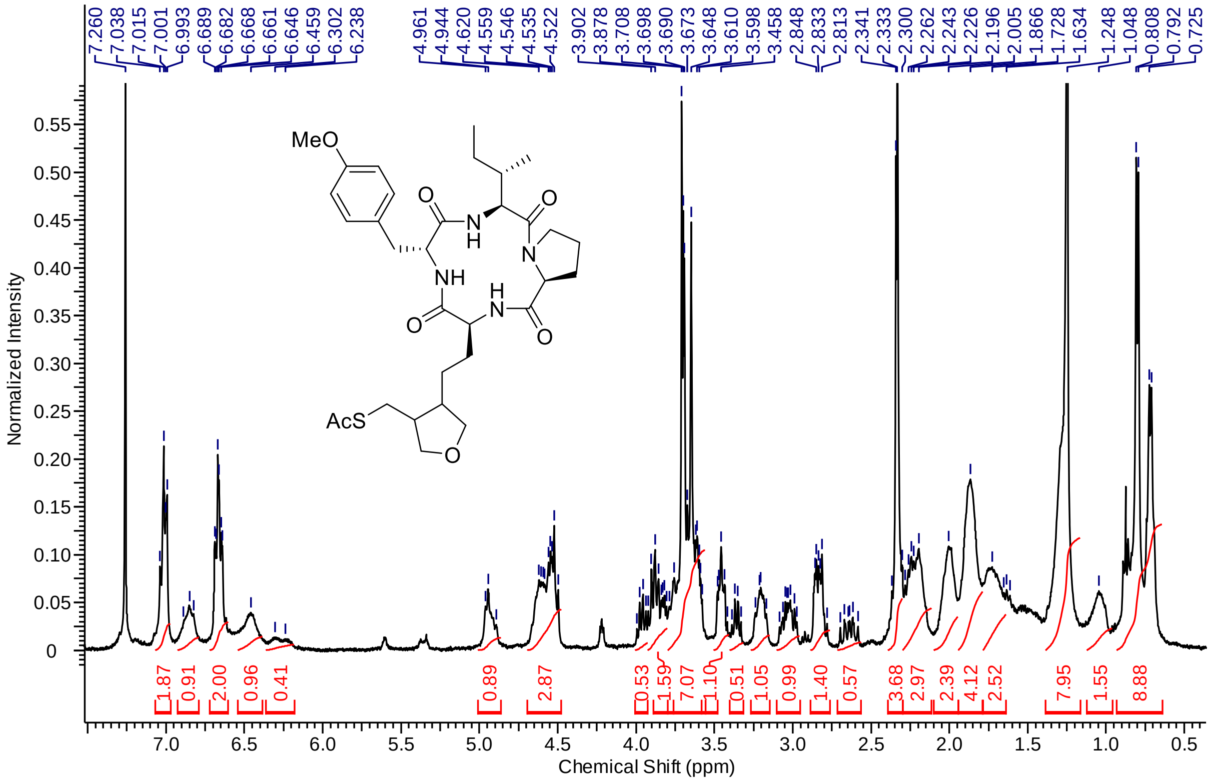

^{13}C NMR (100 MHz, $CDCl_3$)

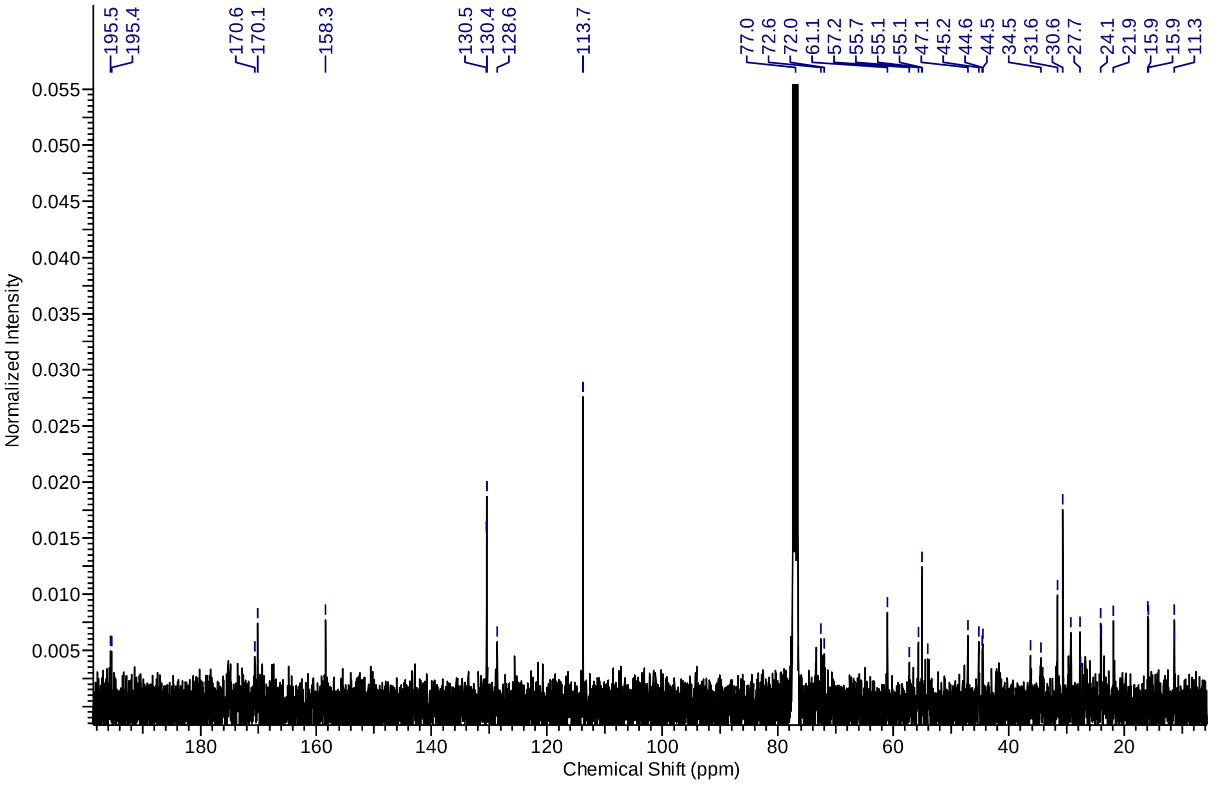

_S_-((4-(2-((3_S_,6_R_,9_S_,14a_S_)-9-((_S_)-_sec_-Butyl)-6-(4-methoxybenzyl)-1,4,7,10-tetraoxotetra-deca-hydropyrrolo[1,2-_a_][1,4,7,10]tetraazacyclododecin-3-yl)ethyl)tetrahydrofuran-3-yl)methyl) benzothioate (14b)

^{1}H NMR (400 MHz, $CDCl_3$)

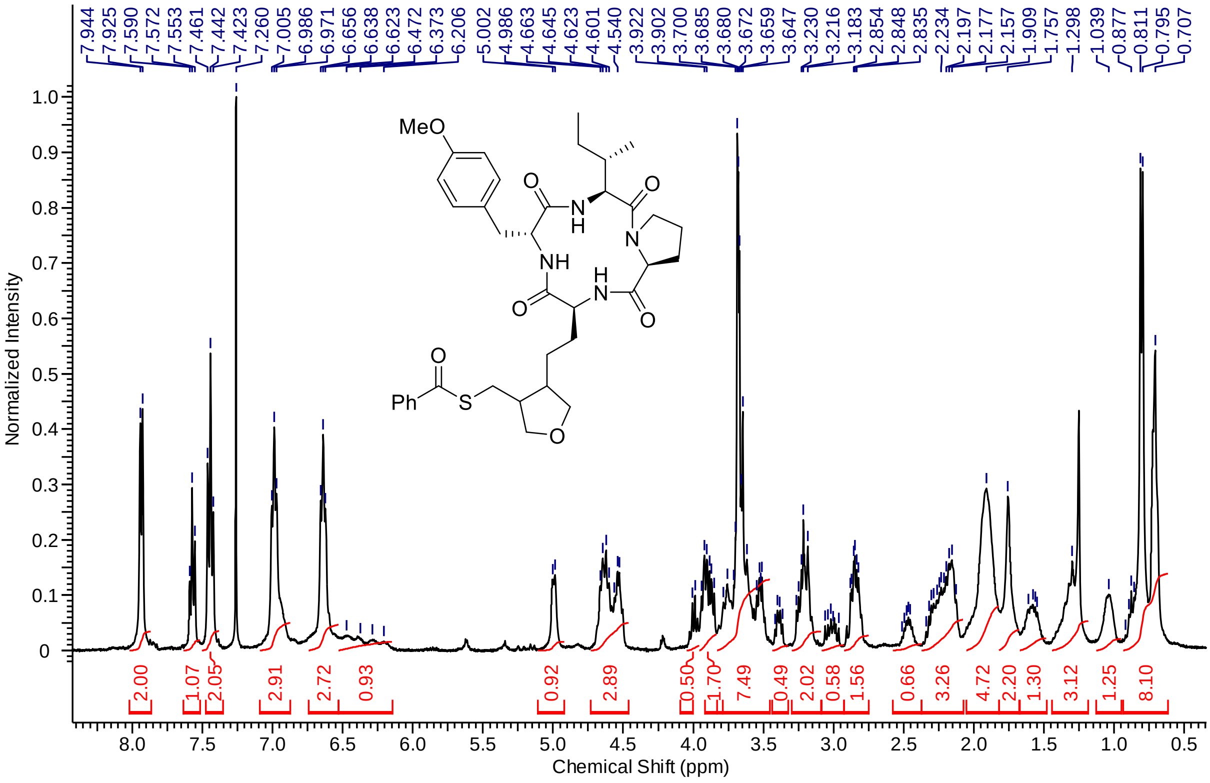

^{13}C NMR (100 MHz, $CDCl_3$)

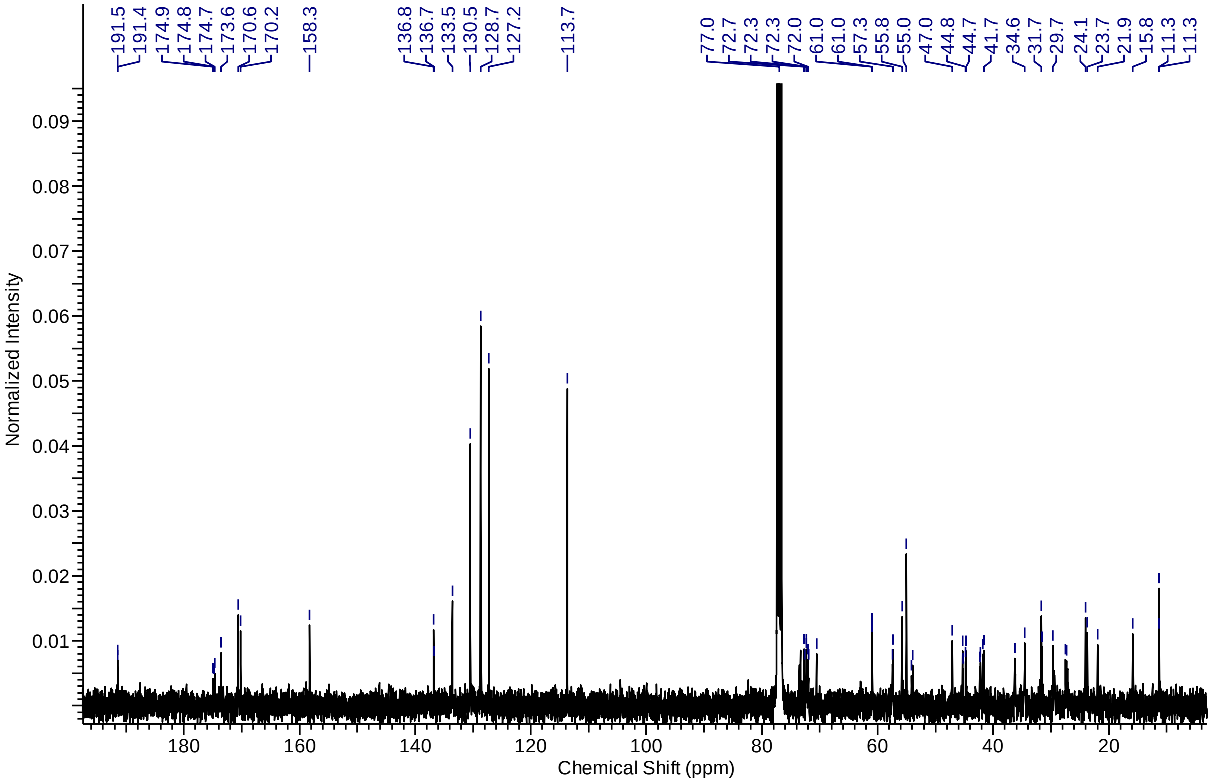

Supplement: File 1 — Detailed synthetic procedures, characterization of all molecules and copies of NMR spectra. [file Beilstein_J_Org_Chem-18-174-s001.pdf]
